# Systemic inflammation, delirium and clinical progression in mild-moderate Alzheimer disease

**DOI:** 10.1016/j.ebiom.2026.106159

**Published:** 2026-02-17

**Authors:** Adam H. Dyer, Helena Dolphin, Laura Morrison, Tara Kenny, Padraic G. Fallon, Colm Cunningham, Antoinette O'Connor, Brian Lawlor, Cliona O'Farrelly, Nollaig M. Bourke, Sean P. Kennelly, NILVAD Study Group

**Affiliations:** a Tallaght Institute for Memory and Cognition, Tallaght University Hospital, Dublin, D24NR0A, Ireland; b Discipline of Medical Gerontology, School of Medicine, Trinity College Dublin, Dublin, D02R590, Ireland; c Trinity Translational Medicine Institute, Trinity Centre for Health Sciences, St. James's Hospital Campus, Dublin, D08W9RT, Ireland; d School of Biochemistry and Immunology, Trinity Biomedical Sciences Institute, Trinity College Dublin, Dublin, D02R590, Ireland; e Academic Unit of Neurology, School of Medicine, Trinity College Dublin, Dublin, D02R590, Ireland; f Global Brain Health Institute, Trinity College Dublin, Dublin, D02XF79, Ireland

**Keywords:** Alzheimer's disease, Plasma, Biomarker, Inflammation, Neuroinflammation, Glial Fibrillary Acidic Protein, Phosphorylated tau-217

## Abstract

**Background:**

Both low-grade systemic inflammation and acute inflammatory events may contribute to Alzheimer Disease (AD) progression. However, studies examining the prognostic utility of systemic inflammatory biomarkers in AD, and how systemic inflammatory events may contribute to clinical trajectories in AD, have yielded conflicting results.

**Methods:**

We quantified plasma cytokines/chemokines in 333 individuals with mild-moderate AD at baseline, 12 and 18 months alongside baseline neurodegenerative biomarkers. AD severity was assessed using the Alzheimer Disease Assessment Scale (ADAS-Cog), Clinical Dementia Rating Scale (CDR-Sb) and Disability Assessment for Dementia (DAD).

**Findings:**

Systemic inflammatory biomarkers were primarily associated with age/socio-demographic characteristics, remained strikingly stable over time, and were not associated with AD progression. Rather, higher baseline plasma p-tau217 was associated with greater yearly progression on both the ADAS-Cog (β: 2.82; 95% CI: 1.12, 4.52; nominal p = 0.001) and DAD (β: −2.34; 95% CI: −3.86, −0.82; nominal p = 0.003). Higher baseline GFAP was also associated with subsequent decline on both the CDR-Sb (β: 1.02; 95% CI: 0.38, 1.67; nominal p = 0.002) and DAD (β: 1.91; 95% CI: −3.45, −0.37; nominal p = 0.02). Experiencing one or more episodes of delirium was associated with accelerated decline on the CDR-Sb at 18-months (β: 2.63; 95% CI: 1.55, 3.71; adjusted p < 0.001).

**Interpretation:**

Biomarkers of neuroinflammation (GFAP), neurodegeneration (p-tau217) and incident delirium, rather than systemic inflammatory biomarkers, were associated with clinically-significant decline in mild-moderate AD.

**Funding:**

10.13039/501100000780European Commission (FP7 grant; 279093); 10.13039/501100004162Meath Foundation (MFRG 121/2021); 10.13039/100010269Wellcome Trust (227946/Z/23/Z & 203930/B/16/Z); Health Research Board (203930/B/16/Z; ECSA-2024-003).

**Research in context**

**Evidence before this study**

Authors performed a thorough literature review through both published (PubMed/CINAHL) and preprint databases in order to identify studies linking systemic inflammatory biomarkers and clinical progression in individuals with dementia due to Alzheimer Disease (AD). Search terms included [“Alzheimer∗” OR “Alzheimer Disease” OR “Dementia”] AND [“Inflammation” OR “Biomarkers”, “Cytokines” OR “Chemokines” OR “Interleukins”] AND [“Blood” OR “Plasma” OR “Serum”]. Only the most relevant citations were selected for inclusion and discussion in this work. Systemic inflammatory biomarkers have consistently shown to be elevated in AD. However, limited studies have evaluated whether systemic inflammatory biomarkers and systemic inflammation drives clinical decline in established AD. Those studies which have yield conflicting results, with many limited by sample size, insensitive assay measurements of circulating chemokines/cytokines and short duration of follow-up.

**Added value of this study**

This work used repeated samples from a well-characterised cohort of individuals living with mild-moderate dementia due to AD to assess longitudinal relationships between systemic inflammatory biomarkers, neurodegenerative biomarkers, systemic inflammatory events and AD progression. Our work demonstrates that biomarkers of neurodegeneration and neuroinflammation as well as incident delirium associates with clinically-meaningful decline in individuals with mild-moderate dementia due to AD. However, systemic inflammatory biomarkers were not associated with subsequent clinical decline.

**Implications of all the available evidence**

Systemic inflammatory biomarkers may be of limited prognostic utility in established AD. Rather, biomarkers of neuroinflammation, neurodegeneration and incident delirium were clinically-significant indicators of future clinical decline in established AD.

## Introduction

There is mounting evidence that a low-grade, sterile pro-inflammatory state—often termed “inflammaging” - is a significant risk factor at population level for the development of Alzheimer Disease (AD).^1^ This is supported by a number of studies demonstrating associations between elevated levels of circulating systemic inflammatory biomarkers–most commonly Interleukin-6 and Tumour Necrosis Factor Alpha (TNF-α)–and the later development of AD.^2^, ^3^, ^4^, ^5^, ^6^, ^7^, ^8^, ^9^, ^10^ Whilst findings are heterogenous, these studies indicate an important role for systemic inflammation either acting as a risk factor for AD or reflecting pathophysiological changes accompanying AD in older adults.

In established AD where prognostic biomarkers are urgently needed, the evidence linking inflammatory biomarkers and AD progression is less clear. Circulating levels of Monocyte Chemoattractant Protein-1 (MCP-1), Eotaxin-1, TNF-α, Interleukin 12p70 (IL-12p70) and Interferon-γ (IFN-γ) have all been linked with accelerated decline in established AD - although findings vary based on study size, assay sensitivity, follow-up duration and clinical assessment.^11^, ^12^, ^13^, ^14^, ^15^ A recent study has demonstrated significant correlations between inflammatory biomarkers and biomarkers of AD pathology, and these correlations associate with worsening decline in AD.^16^ Given the heterogenous and at times conflicting results, there is an urgent need to establish the potential prognostic utility of systemic inflammatory biomarkers in AD.^15^

One of the key unanswered questions in this area is the degree to which systemic inflammatory biomarkers in AD reflect either: i. a systemic response to AD pathology; ii. systemic inflammation as a potentially detrimental risk factor driving further cognitive decline; or iii. reflect sociodemographic/clinical risk factors for AD (such as age, sex or body mass index). Teasing out the associations between AD risk factors, temporal changes in inflammatory biomarkers and subsequently the relationship between these and AD clinical progression is thus an imperative question for the field.

Technological advances have enabled highly-accurate measurement of low-abundant proteins in plasma with increasing multiplexing ability. Crucially, this has firmly established the role of Blood-Based Biomarkers (BBBMs), namely plasma phosphorylated-tau species such as p-tau181 and more so p-tau217 as scalable and accurate diagnostic biomarkers of AD pathology.^17^, ^18^, ^19^ Another BBBM with stable detectability across biofluids is Neurofilament Light (NfL), released when neuronal cells or their axonal membranes are damaged.^20^, ^21^, ^22^ Glial Fibrillary Acidic Protein (GFAP) is another well-performing BBBM which reflects neuro-inflammation and reactive astrogliosis in AD.^23^ GFAP is emerging as an early AD biomarker and predicts conversion to dementia in MCI due to AD.^24^, ^25^, ^26^, ^27^

BBBMs are increasingly being incorporated into diagnostic paradigms in AD.^28^ However, their utility in predicting clinically-meaningful decline in established AD dementia has been less-well studied—but suggest a particular role for p-tau217.^29^ Increasingly, the high-sensitivity platforms employed to measure AD BBBMs are also being used in the study of plasma inflammatory biomarkers—in particular to low-abundant cytokines typically difficult to quantify, for instance TNF-α or IL-12p70—both previously linked to progression in AD.^16^^,^^30^, ^31^, ^32^

In addition to chronic systemic inflammation, there is also evidence that acute episodes of systemic inflammation may accelerate clinical progression in AD. In 300 individuals with AD, Systemic Inflammatory Events (SIEs) —short lived inflammatory insults not directly involving the Central Nervous System (CNS) —were associated with clinical decline in established AD.^12^ This is consistent with a large body of work demonstrating exaggerated CNS immune responses following SIEs in mouse models of neurodegeneration,^33^^,^^34^ with subsequent acceleration of degenerative pathology and functional decline following the insult in mice^35^, ^36^, ^37^ and in non-human primates.^38^ However, human studies linking SIEs and disease progression in AD are rare and replication studies are required to examine whether they link with clinically-meaningful decline in established AD.

Here, we used longitudinal plasma and Cerebrospinal Fluid (CSF) samples from individuals with mild-moderate dementia due to AD followed for 18-months with repeat cognitive and dementia severity assessment. Using high-sensitivity immunoassay technology, we aimed to investigate the prognostic utility of biomarkers of systemic inflammation, neuro-inflammation and neurodegeneration as well as the contribution of SIEs and delirium to clinical progression in established dementia due to AD.

## Methods

### Study setting

NILVAD was a European phase 3 investigator-led, double blind study of the antihypertensive Nilvadipine in mild-moderate dementia due to AD.^39^ In the NILVAD trial, treatment with Nilvadipine had no effect on change in cognitive function/dementia severity over 18 months.^40^ Inclusion criteria included a diagnosis of AD as per the Neurological and Communicative Disorders and Stroke/Alzheimer's Disease (NINCDS-AD) Criteria and a Mini-Mental State Examination score of 12–26.^41^^,^^42^ Individuals with significant neurological/cardiovascular comorbidity, severe psychiatric illness, active substance misuse or renal/liver impairment were excluded from participation.

The NILVAD Study was powered to detect a treatment effect of *Nilvadipine* on AD progression over 78 weeks. The planned sample size of 250 participants per arm provided 90% power to detect a 3.5 difference in the Alzheimer Disease Assessment Scale—Cognitive Subsection (ADAS-Cog) and an 81% change of detecting a corresponding effect on the Clinical Dementia Rating Scale—Sum of Boxes (CDR-Sb), allowing for 30% attrition, as described previously.^40^ The current data consist of a secondary analyses of data from the blood and genetic NILVAD sub-study, described in detail below.

### Participant clinical and dementia assessment

All individuals underwent baseline clinical assessment which ascertained socio-demographic characteristics (including age, sex, body mass index, education) as well as AD characteristics and medical comorbidity (assessed using an adapted version of the Charlson Comorbidity Index for individuals with dementia).^43^ Sex was recorded as male/female based on clinical records; no gender identity or ethnicity data were collected. Medication use was recorded using the Anatomic Therapeutic Classification [ATC] system. Use of Non-Steroidal Anti-Inflammatory Drugs (NSAIDs), including topical use, and Glucocorticoids (including inhaled and oral) were both identified using the relevant ATC codes.

AD cognitive severity was assessed using the ADAS-Cog as the primary endpoint with the co-secondary endpoints of change in dementia severity rated using the CDR-Sb and change in disability due to dementia on the Disability Assessment in Dementia (DAD).^44^, ^45^, ^46^ All three assessments were conducted at baseline and at 12 and 18 month timepoints.

### Adverse events, delirium and systemic inflammatory events in NILVAD

Adverse events were recorded on eight discrete occasions throughout the study duration (weeks 6, 13, 26, 39, 52, 65, 78, and 82 weeks). Any adverse event/severe adverse events were recorded by study assessors as a free-text description—obtained by direct report from the participant, and caregiver report where appropriate. Additionally, caregivers or family members accompanying study participants were interviewed about changes in cognition likely to indicate incident delirium using the Family Confusion Assessment Method (FAM-CAM).^47^

Adverse events free-text logs were subsequently dual-reviewed by physicians in geriatric medicine to identify Systemic Inflammatory Events (SIEs), with any discrepancies adjudicated by a third physician in geriatric medicine. SIEs were defined as: i. any short-lived infection (local/systemic), less than 3 months in duration likely to result in an inflammatory response; ii. any acute systemic inflammatory insult (including pathogen exposure or tissue damage) and iii. any surgery/trauma or other insult likely to result in an inflammatory response.^2^^,^^12^

### Biomarker sub-studies in NILVAD

All individuals participating in NILVAD were eligible to participate in the NILVAD sub-studies with recruitment based on local site.^48^ For the blood and genetic biomarker sub-study, participants donated extra blood (30 mL) at baseline, 12-months and 18-months using standard aseptic venepuncture. From those, 1 mL was used for APOE genotyping at baseline, with the remaining 29 mL processed immediately on site. For plasma, 2 × 10 mL EDTA tubes were centrifuged for 5 min at 1380×*g* and plasma stored in 1 mL aliquots. For the Cerebrospinal Fluid (CSF) sub-study, diagnostic Lumbar Puncture (LP) was performed following local guidelines and aseptic technique using a Spinal Needle Quincke Type Point 0.7 × 75 mm at L3/L4 or L4/L5, with 10 mL CSF collected in a polypropylene tube. CSF was centrifuged at 2000×*g* for 10 min and cell-free CSF aliquoted into 1 mL aliquots and stored at −80 °C locally.

After local storage at −80 °C, samples were transferred on temperature-monitored dry ice to a central biobank repository in Centre Hospitalier Universitaire de Lille, Lille, France for long-term monitored storage at −80 °C for up to 8 years prior to analysis. For the current study, samples were transferred on dry-ice using a temperature-monitored shipment to Trinity Translational Medicine Institute in Dublin, Ireland and continuously stored at −80 °C. For all analyses in the current study, a fresh previously unthawed aliquot of plasma/CSF was used for each biomarker panel-namely cytokine S-PLEX panel/chemokine V-PLEX panel/neurodegenerative S-PLEX panel.

### Measurement of systemic inflammatory biomarkers, neurodegenerative and neuroinflammatory biomarkers

High-sensitivity immuno-assays were used to assess inflammatory chemokines/cytokines. Based on the results of prior literature review, in addition to biological plausibility in AD progression and high-sensitivity immunoassay availability, a final panel of 10 cytokines and chemokines were selected. This included cytokines IFN-γ, IL-6, IL-10, IL-12p70, IL-17A and TNF-α and chemokines, namely Eotaxin, Interferon Gamma Inducible Protein 10 (IP-10), MCP-1 and Macrophage Inflammatory Protein 1β (MIP-1β). The pro-inflammatory cytokines IL-6, TNF-α and IL-12p70^49^^,^^50^ represent classical pro-inflammatory cytokines, deliberately balanced with measurement of IL-10 to capture anti-inflammatory responses. Both IFN-γ and IL-17A reflect T-Cell cytokines, known to be implicated in AD progression.^14^^,^^51^ Meanwhile, MCP-1 is the key monocyte chemoattractant protein and Eotaxin, IP-10 and MIP-1β reflect the Senescence-Associated Secretory Phenotype known to drive brain ageing and important in the pathogenesis of AD, with direct neuromodulatory effects.^11^^,^^52^, ^53^, ^54^, ^55^

Based on required performance characteristics and analyte availability, the Mesoscale Discovery (MSD)™ S-PLEX/V-PLEX platform was used with the six cytokines measured using the ultra-sensitive S-PLEX system (Catalogue No. K1593S) and the four chemokines using the V-PLEX system (Catalogue No. K15047D). The S-PLEX system is an ultra-sensitive Electro-Chemiluminescence^56^ assay system which reduce the lower limit of detection compared to conventional immune-assays by 10–1000 fold enabling it to accurately measure low-abundant proteins in biological samples.^57^^,^^58^ For neurodegenerative biomarkers, phosphorylated-tau 181 (p-tau181), phosphorylated-tau 217 (p-tau217), total-tau (t-tau), Glial Fibrillary Acidic Protein (GFAP) and Neurofilament Light (NfL) were assessed using the S-PLEX platform (Catalogue No. K151AGMS/K151APFS/K15639S respectively). Inflammatory chemokines and cytokines were assessed at baseline, 12-months and 18-months in plasma samples and at baseline and 18-month CSF samples. The five neurodegenerative biomarkers were assessed in baseline samples only.

For measurement of biomarkers, manufacturer's instructions were followed for S-PLEX/V-PLEX kits. Standards were provided with each individual plate and reconstituted following manufacturer's protocols. Having completed assays as per manufacturer's instructions, plates were read using the MSD Quick Plex™ 120 mm plate reader instrument. Data for each plate was exported to the MSD Discovery Workbench v4.0 software, where a 5-parameter logistic regression curve was applied to interpolate unknown values from the 4-fold serial dilution standard curve supplied with each kit. For measurement of inflammatory chemokines/cytokines, baseline, 12-month and 18-month samples were always measured on the same plate to minimise the impact of intra-assay variation. A plasma pool was created by pooling plasma samples from 12 NILVAD donors selected to represent the extremes of age, education and BMI within the cohort with an equal distribution of males and females. This pool was assessed across each assay plate to calculate intra-assay Coefficient of Variation (CV). The inter-assay CV was calculated from duplicate samples on the same plate.

### Statistics

Descriptive statistics were used to summarise clinical and demographic information, with the approach varying according to data type and distribution. Continuous, normally distributed data were described using means and Standard Deviation (SD) and data that violate the normality distribution with medians and Interquartile Ranges (IQR) as appropriate. Categorical data were reported as proportions and percentages. Between-group comparisons used t-tests, chi-square tests and Wilcoxon rank sum tests as appropriate. Data were assessed for normality in the first instance using histograms, Q–Q plots and Shapiro–Wilk tests. As all biomarkers demonstrated strong right skew, biomarker concentrations were first natural-log transformed and natural-log transformed concentrations examined for normality. To aid interpretation, these natural-log transformed values were then z-scored across the cohort.

In examining baseline associations between clinical factors and inflammatory/neurodegenerative biomarker concentration, univariate linear regression was first applied without adjustments. Given the exploratory nature of this analysis, the Benjamin-Yekutieli method was used to control for the False Discovery Rate (FDR) and correct for multiple testing. Results are presented as beta coefficients (β) followed by 95% confidence intervals (CI), corresponding unadjusted (“p”) or adjusted p-values (“p adj”). These analyses identified the potential relationships between baseline demographic/AD severity characteristics and biomarker levels.

To subsequently investigate biomarkers predicting changes over time, mixed-effects linear regression modelling was used. For each clinical outcome (ADAS-Cog, CDR-Sb and DAD—as the dependent variable), we fitted linear mixed-effects models with random intercepts and slopes to account for repeated measures within participants. Time (years since baseline) was treated as a continuous variable. Each biomarker level (natural-log transformed and z-scored) was included as a *Biomarker∗Time* predictor (dependent variable) to assess the impact of baseline biomarker on subsequent decline (ADAS-Cog, CDR-Sb and DAD). As above, natural-log transformed, and z-scored biomarker values were used which were normally distributed. Results for these models are reported for the interaction term as β coefficients with 95% confidence intervals and p-values. All models were initially run unadjusted. Where multiple predictors were examined at once (for instance, baseline values of each of the ten chemokines/cytokines and subsequent cognitive outcome), multiple testing was again applied using the Benjamin-Yekutieli method and adjusted p-values reported. For predictors in change of inflammatory biomarkers over time, the same method was used, except with change in inflammatory biomarker concentration (natural-log transformed and z-scored) as the dependent variable and clinical predictor as the independent variable, again with multiple testing correction applied.

Where significant in univariate analysis after multiple testing adjustment, associations were then adjusted for age, sex, body mass index, education, baseline severity score (ADAS-Cog, CDR-Sb, DAD), comorbidity and study group (*Nilvadipine* vs placebo) and adjusted β coefficients, 95% CI and p-values reported. For baseline severity score, where ADAS-Cog was used as the dependent variable, baseline ADAS-Cog was used to adjust for baseline dementia severity, where CDR-Sb was the dependent variable, baseline CDR-Sb used to adjust for baseline severity and the same for DAD. For baseline biomarkers significantly associated with subsequent clinical decline, results were individually re-analysed using biomarker tertile as the independent variable, to aid interpretability of findings and assess the potential clinical relevance of findings, with mixed-effects model effect sizes and nominal p-values reported.

To account for the fact that AD diagnosis at time of recruitment in the current study was based on clinical criteria only (i.e no biomarker measurement), sensitivity analyses was conducted for significant associations to only include participants with a high likelihood of AD pathology. This was based on a pre-established and published cut-off in our lab of 6.01 pg/mL for plasma p-tau217 which was calculated based on a 97.5% specificity cut-off for AD pathology (CSF defined amyloidosis).^59^ At this threshold, a positive plasma p-tau217 result has a Positive Predictive Value of 96.61% for AD pathology.^59^

For associations between clinical events (adverse events, severe adverse events, SIEs, hospitalisations, delirium) and change in cognitive/dementia severity, linear regression was used with change from baseline in ADAS-Cog/CDR-Sb/DAD as the dependent variable and events as the independent variable. Again univariate analyses were adjusted for multiple testing using the Benjamin-Yekutieli method and additionally adjusted for age, sex, body mass index, education, baseline dementia severity (on either ADAS-Cog, CDR-Sb or DAD as above), diagnosis duration, comorbidity and study group.

For all linear models, assumptions were checked by inspection of residual vs fitted plots (examining for linearity and homoscedasticity), Q–q plots of residuals and random effects (for normality). No major violations were observed. All data analyses and visualisation were carried out using STATA v17.0 (STATACorp LLC, Texas, USA) and R-Studio (Version 2024.04.2+764). This observational analysis is reported in accordance with the STROBE guidelines.

### Ethics

Ethical approval was granted from appropriate National Competent Authorities, Independent Ethics Committees and Institutional Review Boards for all 23 sites. A list of these is provided in the Supplementary Material (Table S1). Written informed consent was obtained from all participants and the NILVAD study. Caregivers provided written consent to accompany the patient to assessments, supervise administration of the study drug and participate in assessments requiring caregiver input.^39^ The trial adhered to the Declaration of Helsinki and International Conference on Harmonisation of Good Clinical Practice (ICH GCP) guidelines at the time of study conduct.^40^

### Role of funders

The funders had no role in the study design, collection or analysis of data, interpretation of results, writing or drafting of manuscript or decision to publish.

## Results

### Participant characteristics

Of 510 individuals with mild-moderate AD dementia participating in NILVAD, 333 participated in the blood and genetic sub-study and had a baseline plasma sample available for analysis. Overall, individuals participating in the blood sub-study had a mean age of 72.8 (±8.3) years and more than half (62.2%) were female. Median time since AD diagnosis was 1.1 years (IQR: 0.5–2.3 years). Consistent with mild-moderate dementia, mean baseline cognitive performance on the ADAS-Cog was 34.5 (±10.6), dementia severity on the CDR-Sb was 5.3 (±2.8) and mean disability assessed using the DAD was 29.3 (±8.0).

Amongst participants in the NILVAD blood and genetics sub-study, treatment with Nilvadipine, assessed by an Arm (Nilvadipine vs Placebo) X Time (in months) interaction, was not associated with clinical progression on the ADAS-Cog (β: −0.03; −0.50, 0.43; p = 0.86; mixed-effects linear regression), CDR-Sb (β: 0.01; −0.03, 0.05; p = 0.60), or DAD (β: 0.01; −0.09, 0.11; p = 0.85; mixed-effects linear regression). Data is visualised in Figure S1. Thus, in the current analysis, treatment arms were pooled for subsequent biomarker analysis.

Of the 333 individuals in the blood and genetic bio-marker sub-study with a plasma sample at baseline, all had complete clinical data available at baseline including scores on the ADAS-Cog, CDR-Sb and DAD. By 12 months, 279 had a repeat blood sample taken, of whom seven were unable to complete the ADAS-Cog. All 279 had complete CDR-Sb/DAD assessment at 12 months. At 18 months, 258 had a repeat blood sample taken, of whom 13 were unable to complete the ADAS-Cog, with all 258 having a repeat assessment on the CDR-Sb and DAD. Of the 333 participants, there were three deaths during the study period, all of whom had a baseline sample only and no further assessment/blood sampling.

For the CSF sub-study, 93 had a LP performed at baseline and 55 had a repeat LP at 18-months. Baseline characteristics of those participating in both sub-studies are provided in Table 1, alongside the characteristics of the overall NILVAD cohort. Of note, individuals in the CSF sub-study were younger (t = 3.6, p < 0.001). There were no other statistically significant differences in baseline demographic or AD characteristics between either sub-study groups and the overall NILVAD cohort.

**Table 1:** Baseline characteristics of study participants in the overall NILVAD cohort, blood and CSF sub-studies.

	NILVAD cohort (N = 510)	Blood sub-study (N = 333)	CSF sub-study (N = 93)
Age (Years), Mean (SD)	74.1 (8.3)	72.4 (8.3)	69.5 (8.4)
Sex			
Female, n (%)	316 (62.0%)	207 (62.2%)	49 (53.3%)
Male, n (%)	194 (38.0%)	126 (37.8%)	44 (47.3%)
Body Mass Index (kg/m^2^), Mean (SD)	25.4 (4.3)	25.6 (4.1)	26.0 (4.0)
Education, Age Finished, Median (IQR)	16 (14–18)	16 (14–19)	16 (14–20)
Adjusted Comorbidity Index, Median (IQR)	0 (0–1)	0 (0–1)	0 (0–1)
Diagnosis Duration (Years), Median (IQR)	1.0 (0.5–2.3)	1.0 (0.4–2.2)	0.6 (0.2–1.7)
NSAID Use (Any), n (%)	36 (7.1%)	27 (8.1%)	6 (6.5%)
Glucocorticoid Use (Any), n (%)	23 (4.5%)	12 (3.6%)	2 (2.2%)
Alzheimer Disease Severity			
ADAS-Cog (Total), Mean (SD)	34.5 (10.6)	34.8 (10.8)	35.0 (10.4)
CDR-Sb (Total), Mean (SD)	5.3 (2.8)	5.3 (2.8)	5.3 (2.3)
DAD (Total), Mean (SD)	29.1 (8.0)	29.3 (8.0)	29.5 (6.8)
Study Group			
Nilvadipine, n (%)	252 (49.4%)	163 (49.0%)	41 (44.6%)
Placebo, n (%)	258 (50.6%)	170 (51.1%)	51 (55.4%)

SD: Standard Deviation; IQR: Interquartile Range; ADAS-Cog: Alzheimer Disease Assessment Scale—Cognitive Subsection; CDR-Sb: Clinical Dementia Rating—Sum of Boxes; DAD: Disability Assessment for Dementia.

### High-sensitivity measurement of biomarkers

Analysis of plasma biomarkers was carried out as described above. Using optimised high-sensitivity assays, detectable levels of all ten cytokines/chemokines were obtained for >99% of plasma samples tested (Performance characteristics are given in Table S2). In CSF, all cytokines/chemokines were detectable in >95% of tested samples apart from IL-17A (detectable in 93.7%) and IL-12p70 (detectable in 75.6%) (Table S2). For the five neurodegenerative and neuroinflammatory biomarkers measured in plasma and CSF at baseline only, levels were detectable for all samples (Table S3).

### Baseline plasma systemic inflammatory biomarkers largely reflect age and sociodemographic characteristics in mild-moderate AD

**Fig. 1:**
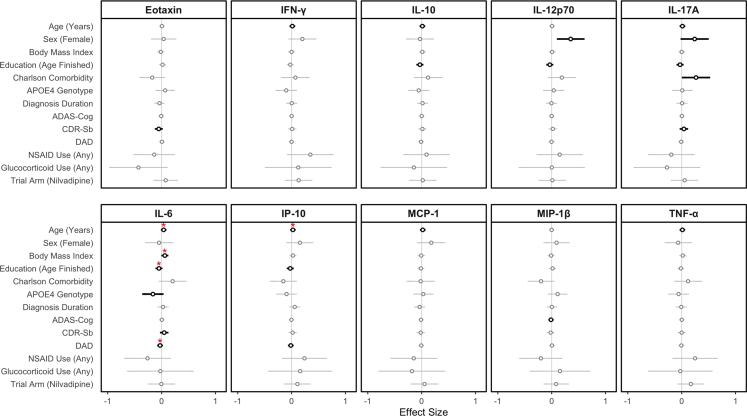
**Associations Between Participant Characteristics and Baseline Plasma Chemokine/Cytokine Concentration.** Results of linear regression are presented as Beta Coefficients (Circles) and 95% Confidence Intervals (Lines). Non-significant results are coloured grey, with significant associations coloured black (unadjusted). N = 333. Multiple-testing adjusted p-values are provided as red asterisks where ∗p < 0.05, ∗∗p < 0.01, ∗∗∗p < 0.001. ADAS-Cog: Alzheimer Disease Assessment Scale—Cognitive Subsection; CDR-Sb: Clinical Dementia Rating—Sum of Boxes; DAD: Disability Assessment for Dementia; IFN-γ: Interferon Gamma; IL-10: Interleukin 10; IL-12p70: Interleukin 12p70; IL-17A: Interleukin 17A; IL-6: Interleukin 6; IP-10: Interferon-gamma-Inducible Protein 10; MCP-1: Monocyte Chemoattractant Protein 1; MIP-1β: Macrophage Inflammatory Protein 1 Beta; TNF-α: Tumour Necrosis Factor Alpha.

We first aimed to establish associations between both (i) demographic/AD risk factors and (ii) AD characteristics (disease severity, duration etc.) and baseline plasma chemokine/cytokine concentrations. Increasing age was associated with greater levels of seven of the studied analytes, with those between increasing age and greater IL-6/greater IP-10 persisting following multiple testing adjustment (β: 0.04; 95% CI: 0.01, 0.03; adj. p < 0.001/β: 0.02; 95% CI: 0.01, 0.04; adj. p = 0.02 respectively; linear regression). Sex, education and BMI were significantly associated with levels of several analytes (Fig. 1), with associations between greater BMI/lower levels of educational attainment and greater plasma IL-6 persisting following adjustment for multiple testing (β: 0.06; 95% CI: 0.01, 0.03; adj. p = 0.001/β: −0.05; 95% CI: −0.07, −0.02; adj. p = 0.03 respectively; linear regression). With regards to AD characteristics, increasing IL-6 was associated with greater disability on the DAD (β: −0.03; 95% CI: −0.04, −0.01; adj. p = 0.01; linear regression). In a multivariate model incorporating age, fewer years of education, lower BMI and greater disability (DAD) as predictors, all were independently associated with plasma IL-6 (all p < 0.001; linear regression).

### Baseline plasma inflammatory biomarkers do not predict accelerated disease progression in mild-moderate AD

Next, we examined whether baseline concentrations of plasma cytokines/chemokines were associated with subsequent clinical progression and cognitive decline in AD. Notably, in NILVAD the study drug (Nilvadipine) was not associated with clinical progression over 18-months. The mean decline for those in the blood sub-study over 18-months was +8.9 (±8.8) on the ADAS-Cog, +3.4 (±3.3) on the CDR-Sb and −7.7 (±8.1) on the DAD. There were no significant associations between baseline concentration of any of the plasma chemokine/cytokine and subsequent decline on any of the three outcome measures assessed (Table 2).

**Table 2:** Baseline plasma cytokine and chemokine concentration and subsequent cognitive decline and clinical progression over 18 Months in mild-moderate Alzheimer disease.

Outcome	Predictor	β	Lower CI	Upper CI	p-value (nominal)
Change in ADAS-Cog	*Plasma IFN-γ∗Time*	−0.05	−0.28	0.17	0.65
	*Plasma IL-10∗Time*	−0.01	−0.24	0.21	0.91
	*Plasma IL-12p70∗Time*	0.00	−0.22	0.23	0.98
	*Plasma IL-17A∗Time*	0.01	−0.23	0.24	0.96
	*Plasma IL-6∗Time*	0.01	−0.21	0.23	0.92
	*Plasma TNF-α∗Time*	−0.06	−0.29	0.17	0.61
	*Plasma Eotaxin∗Time*	0.00	−0.24	0.24	0.99
	*Plasma IP-10∗Time*	−0.06	−0.30	0.17	0.60
	*Plasma MCP-1∗Time*	−0.04	−0.28	0.20	0.73
	*Plasma MIP1-β∗Time*	−0.01	−0.25	0.24	0.95
Change in CDR-Sb	*Plasma IFN-γ∗Time*	0.00	−0.08	0.09	0.94
	*Plasma IL-10∗Time*	0.02	−0.06	0.11	0.60
	*Plasma IL-12p70∗Time*	−0.01	−0.09	0.08	0.90
	*Plasma IL-17A∗Time*	0.03	−0.05	0.12	0.45
	*Plasma IL-6∗Time*	−0.02	−0.11	0.07	0.66
	*Plasma TNF-α∗Time*	−0.05	−0.14	0.04	0.27
	*Plasma Eotaxin∗Time*	−0.02	−0.11	0.07	0.68
	*Plasma IP-10∗Time*	−0.04	−0.13	0.05	0.39
	*Plasma MCP-1∗Time*	−0.01	−0.10	0.08	0.89
	*Plasma MIP1-β∗Time*	0.00	−0.08	0.09	0.94
Change in DAD	*Plasma IFN-γ∗Time*	0.02	−0.18	0.21	0.88
	*Plasma IL-10∗Time*	0.07	−0.12	0.27	0.47
	*Plasma IL-12p70∗Time*	0.01	−0.19	0.21	0.91
	*Plasma IL-17A∗Time*	0.05	−0.15	0.25	0.63
	*Plasma IL-6∗Time*	0.03	−0.17	0.23	0.80
	*Plasma TNF-α∗Time*	0.01	−0.19	0.22	0.88
	*Plasma Eotaxin∗Time*	0.05	−0.16	0.25	0.66
	*Plasma IP-10∗Time*	0.04	−0.17	0.25	0.70
	*Plasma MCP-1∗Time*	0.02	−0.18	0.23	0.82
	*Plasma MIP1-β∗Time*	−0.09	−0.30	0.13	0.42

Results are presented as Beta Coefficients (β) and 95% Confidence Intervals (CI) from mixed-effects linear regression with change in cognitive score/dementia severity rating as the dependent variable and an interaction term between log-transformed, z-scored chemokine/cytokine concentration at baseline and time (in years). Models are unadjusted. ADAS-Cog: Alzheimer Disease Assessment Scale—Cognitive Subsection; CDR-Sb: Clinical Dementia Rating—Sum of Boxes; DAD: Disability Assessment for Dementia; IFN-γ: Interferon Gamma; IL-10: Interleukin 10; IL-12p70: Interleukin 12p70; IL-17A: Interleukin 17A; IL-6: Interleukin 6; IP-10: Interferon-gamma-Inducible Protein 10; MCP-1: Monocyte Chemoattractant Protein 1; MIP-1β: Macrophage Inflammatory Protein 1 Beta; TNF-α: Tumour Necrosis Factor Alpha.

### Systemic inflammatory biomarkers remain remarkably stable despite clinical progression in mild-moderate AD

**Fig. 2:**
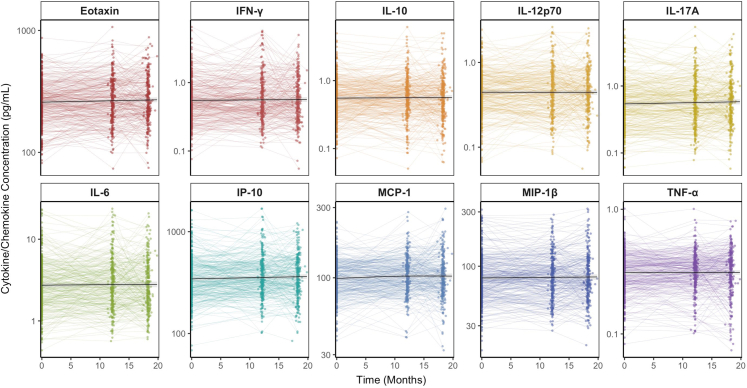
**Longitudinal Trajectories in Plasma Chemokine/Cytokine Concentration.** Plasma cytokine/chemokine concentrations (in pg/mL) are graphed across baseline, 12 and 18 month study visits alongside line of best fit (and 95% confidence interval) for each chemokine/cytokine over time. Concentrations are given in pg/mL on a log scale. N = 333 at baseline, 279 at 12-Months, 258 at 18-Months; IFN-γ: Interferon Gamma; IL-10: Interleukin 10; IL-12p70: Interleukin 12p70; IL-17A: Interleukin 17A; IL-6: Interleukin 6; IP-10: Interferon-gamma-Inducible Protein 10; MCP-1: Monocyte Chemoattractant Protein 1; MIP-1β: Macrophage Inflammatory Protein 1 Beta; TNF-α: Tumour Necrosis Factor Alpha.

Plasma chemokine and cytokines were additionally quantified at 12 and 18-month timepoints. There were no significant changes in any plasma chemokine or cytokine levels despite clinically-meaningful decline in AD (Fig. 2). In analysis examining predictors of cytokine levels, neither time (in months) nor assignment to study drug (Nilvadipine) were associated with any effect on longitudinal plasma chemokine/cytokine trajectories (Figure S2/S3). Similarly, AD severity or disease characteristics were not associated with longitudinal chemokine/cytokine trajectories (Figure S2/S3). There were no significant correlations observed between change in z-scored, natural log-transformed cytokine concentration and AD progression (change in ADAS-Cog, CDR-Sb or DAD) at either 12-months (Figure S4) or 18-months (Figure S5).

### Determinants of neurodegenerative/neuroinflammatory plasma biomarker concentrations in mild-moderate AD

**Fig. 3:**
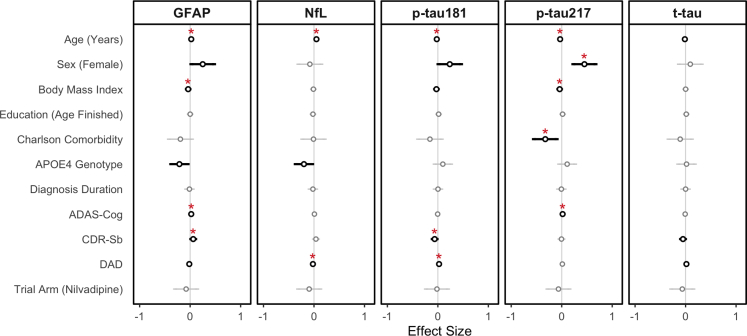
**Associations Between Participant Characteristics and Baseline Plasma Neurodegenerative/Neuroinflammatory Biomarker Concentration.** Results of linear regression are presented as Beta Coefficients (Circles) and 95% Confidence Intervals (Lines). Non-significant results are coloured grey, with significant associations coloured black (unadjusted). N = 333. Multiple-testing adjusted p-values are provided as red asterisks where ∗p < 0.05, ∗∗p < 0.01, ∗∗∗p < 0.001. ADAS-Cog: Alzheimer Disease Assessment Scale—Cognitive Subsection; CDR-Sb: Clinical Dementia Rating—Sum of Boxes; DAD: Disability Assessment for Dementia; GFAP: Glial Fibrillary Acidic Protein; p-tau217: Phosphorylated Tau 217; NfL: Neurofilament Light; p-tau181: Phosphorylated Tau 181; t-tau: Total Tau.

Next, we examined the associations between sociodemographic characteristics/AD characteristics and baseline neuroinflammatory (GFAP) and neurodegenerative (p-tau181, p-tau217, NfL, t-tau) biomarkers. After multiple testing adjustment, age was associated with greater levels of baseline plasma GFAP (β: 0.02; 95% CI: 0.01, 0.03; adj. p = 0.03; linear regression), greater NfL (β: 0.05; 95% CI: 0.01, 0.04; adj. p = 0.01; linear regression), lower p-tau181 (β: −0.02; 95% CI: −0.03, 0.01; adj. p = 0.04; linear regression) and lower p-tau217 (β: −0.03; 95% CI: −0.04, −0.02; adj. p = 0.02; linear regression). Female sex was associated with greater p-tau217 (β: 0.45; 95% CI: 0.23, 0.69; adj. p = 0.01; linear regression) and increasing BMI with lower levels of both plasma GFAP (β: 0.45; 95% CI: 0.23, 0.69; adj. p = 0.03; linear regression) and p-tau217 (β: −0.04; 95% CI: −0.07, −0.01; adj. p = 0.02; linear regression). Greater comorbidity was associated with lower p-tau217 (β: −0.33; 95% CI: −0.55, −0.10; adj. p = 0.03; linear regression) (Fig. 3).

For AD severity, greater levels of both p-tau217 and GFAP were associated with poorer cognitive performance on the ADAS-Cog (β: 0.02; 95% CI: 0.01, 0.03; adj. p = 0.01/β: −0.04; 95% CI: −0.07, −0.01; adj. p = 0.01 respectively; linear regression). GFAP was associated with greater dementia severity on the CDR-Sb (β: 0.06; 95% CI: 0.02, 0.10; adj. p = 0.05; linear regression). Greater NfL was associated with greater disability due to AD (β: −0.02; 95% CI: −0.03, −0.01; adj. p = 0.04; linear regression). The opposite was seen for plasma p-tau181 than for other biomarkers with greater p-tau181 being associated with lower CDR-Sb and greater DAD (indicating less disability) scores respectively. Results are visualised in Fig. 3.

### Baseline p-tau217 and GFAP are associated with subsequent decline in mild-moderate AD

**Fig. 4:**
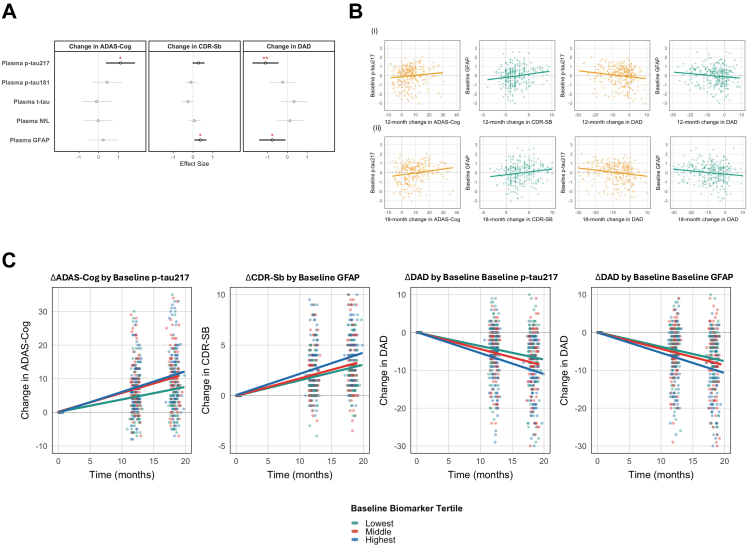
**Associations Between Baseline Neurodegenerative/Neuroinflammatory Biomarker Concentration and Subsequent Cognitive Decline (N** = **333).** (A) Results of linear regression (with natural-log transformed z-scored baseline biomarker concentration∗time as the independent variable and change in cognitive test score/dementia severity the dependent variable) are presented as unadjusted Beta Coefficients (Circles) and 95% Confidence Intervals (Lines). Non-significant results are coloured grey, with significant associations coloured black. Multiple-testing adjusted p-values are provided as red asterisks where ∗p < 0.05, ∗∗p < 0.01, ∗∗∗p < 0.001. (B) Biomarkers passing the threshold for multiple testing correction are visualised against change in cognitive score at (i) 12 months and (ii) 18 months study visits. (C) The relationship between change in cognitive score/dementia severity and time is graphed by tertile of baseline biomarker concentration for those associations that persisted on multiple testing adjustment. ADAS-Cog: Alzheimer Disease Assessment Scale—Cognitive Subsection; CDR-Sb: Clinical Dementia Rating—Sum of Boxes; DAD: Disability Assessment for Dementia. GFAP: Glial Fibrillary Acidic Protein; p-tau217: Phosphorylated Tau 217; NfL: Neurofilament Light; p-tau181: Phosphorylated Tau 181; t-tau: Total Tau.

Subsequently, the association between baseline neurodegenerative markers and subsequent clinical progression was examined. After adjustment for multiple testing, increasing baseline plasma p-tau217 was significantly associated with subsequent cognitive decline on the ADAS-Cog (β: 1.11; 95% CI: 0.41, 1.80; adj. p = 0.01; mixed-effects linear regression) and greater AD progression on the DAD (β: −1.11; 95% CI: −1.73, 0.05; adj. p = 0.01; mixed-effects linear regression) (Fig. 4A and B). Meanwhile greater baseline plasma GFAP was significantly associated with greater dementia progression on both the CDR-Sb (β: 0.37; 95% CI: 0.11, 0.64; adj. p = 0.03; mixed-effects linear regression) and the DAD (β: −0.76; 95% CI: −1.39, −0.13; adj. p = 0.04; mixed-effects linear regression). These associations persisted on adjustment for age, sex, BMI, educational attainment, medical comorbidity, baseline severity score on the ADAS-Cog/CDR-Sb/DAD and study group (*Nilvadipine* vs. placebo) for both baseline p-tau217 (β: 1.07; 95% CI: 0.48, 1.66; p < 0.001 for ADAS-Cog; β: −1.09; 95% CI: −1.59, −0.59; p = 0.003 for the DAD; mixed-effects linear regression) and GFAP (β: 0.38; 95% CI: 0.15, 0.61; p = 0.001 for CDR-Sb; β: −0.83; 95% CI: −1.34, −0.32; adj. p = 0.001 for the DAD; mixed-effects linear regression) and subsequent decline.

To aid interpretability of these findings, we performed analysis by tertile of biomarker for both plasma p-tau217 and GFAP. Compared to being in the lowest tertile, being in both the middle and highest tertile of plasma p-tau217 was associated with greater yearly decline on the ADAS-Cog (β: 2.46; 95% CI: 0.77, 4.15; nominal p = 0.004 for middle vs lowest/β: 2.82; 95% CI: 1.12, 4.52; nominal p = 0.001 for highest vs lowest; mixed-effects linear regression). Being in the highest vs lowest tertile of p-tau217 was associated with significant yearly progression on the DAD (β: −2.34; 95% CI: −3.86, −0.82; nominal p = 0.003; mixed-effects linear regression). For baseline GFAP, concentrations in the highest tertile (vs lowest) were associated with greater progression on the CDR-Sb (β: 1.02; 95% CI: 0.38, 1.67; nominal p = 0.002; mixed-effects linear regression) and the DAD (β: 1.91; 95% CI: −3.45, −0.37; nominal p = 0.02; mixed-effects linear regression). Results are visualised is Fig. 4C.

Again, associations persisted on covariate adjustment as above for middle/highest tertile of p-tau217 and greater yearly decline ADAS-Cog (β: 2.38; 95% CI: 0.93, 3.82; nominal p = 0.001/β: 2.68; 95% CI: 1.25, 4.12; nominal p < 0.001 respectively; mixed-effects linear regression) and for highest tertile of p-tau217 and yearly decline on the DAD (β: −2.27; 95% CI: −3.49, −1.03; nominal p < 0.001; mixed-effects linear regression). Under adjusted models, the highest tertile of GFAP was associated with greater yearly decline on the CDR-Sb (β: 1.01; 95% CI: 0.44, 1.58; nominal p = 0.001; mixed-effects linear regression) and DAD (β: −1.99; 95% CI: −3.23, −0.75; nominal p = 0.002; mixed-effects linear regression).

Finally, we performed sensitivity analysis to account for the fact that diagnosis in the NILVAD study was based on clinical criteria which did not incorporate AD biomarkers. Using an in-house cut-off as previously defined for plasma p-tau217 (6.01 pg/mL on MSD S-PLEX assay) with 97.5% specificity for pathologically-defined AD, we re-ran analyses excluding those with a p-tau217 value below this cut-off. 91.2% of participants had a plasma p-tau217 above the cut-off indicating likely AD pathology. After exclusion of those with a “negative” plasma p-tau217 result (below the cut-off), all associations above remained significant and are included in full in Table 3.

**Table 3:** Sensitivity Analysis of Associations Between Baseline Plasma p-tau217/Plasma GFAP and Subsequent Cognitive Decline.

Outcome	Predictor	Unadjusted				Adjusted			
		β	Lower CI	Upper CI	p (nominal)	β	Lower CI	Upper CI	p (nominal)
Change in ADAS-Cog	*Plasma p-tau217∗Time*	1.66	0.71	2.60	0.001	1.56	0.75	2.37	0.001
	Plasma p-tau217 Tertile								
	*Lowest Tertile∗Time*	–	–	–	–	–	–	–	–
	*Middle Tertile∗Time*	2.70	0.90	4.51	0.003	2.56	1.01	4.12	0.001
	*Highest Tertile∗Time*	3.06	1.25	4.87	0.001	2.87	1.32	4.42	0.001
Change in CDR-Sb	*Plasma GFAP∗Time*	0.40	0.11	0.68	0.006	0.41	0.15	0.66	0.002
	Plasma GFAP Tertile								
	*Highest Tertile∗Time*	–	–	–	–	–	–	–	–
	*Middle Tertile∗Time*	0.34	−0.35	1.04	0.329	0.26	−0.36	0.88	0.409
	*Lowest Tertile∗Time*	1.07	0.38	1.76	0.002	1.05	0.44	1.67	0.001
Change in DAD	*Plasma p-tau217∗Time*	−1.49	−2.33	−0.65	0.001	−1.46	−2.15	−0.77	0.001
	Plasma p-tau217 Tertile								
	*Highest Tertile∗Time*	–	–	–	–	–	–	–	–
	*Middle Tertile∗Time*	−1.13	−2.76	0.50	0.175	−1.12	−2.46	−0.21	0.10
	*Lowest Tertile∗Time*	−2.31	−3.94	−0.68	0.006	−2.22	−3.55	−0.90	0.001
	*Plasma GFAP∗Time*	−0.81	−1.47	−0.15	0.017				
	Plasma GFAP Tertile								
	*Highest Tertile∗Time*	–	–	–	–	–	–	–	–
	*Middle Tertile∗Time*	−0.68	−2.29	0.94	0.413	−0.37	−1.69	0.94	0.576
	*Lowest Tertile∗Time*	−1.91	−3.53	−0.30	0.020	−1.97	−3.27	−0.67	0.003

Results of mixed-effects linear regression are presented. Analyses of significant associations between plasma p-tau217/GFAP and cognitive decline were conducted including only those with high-likelihood of Alzheimer's Disease pathology based on plasma p-tau217. Adjusted models included adjustment for age, sex, body mass index, education, baseline AD severity, comorbidity and study group. ADAS-Cog: Alzheimer Disease Assessment Scale—Cognitive Subsection; CDR-Sb: Clinical Dementia Rating—Sum of Boxes; DAD: Disability Assessment for Dementia; GFAP: Glial Fibrillary Acidic Protein; p-tau217: Phosphorylated Tau 217.

### CSF inflammatory and neurodegenerative biomarkers in mild-moderate AD

Analyses were repeated as above for CSF concentrations of all 15 inflammatory and neurodegenerative biomarkers. On examining determinants of baseline CSF inflammatory biomarker associations, only one finding persisted on multiple testing−namely greater levels of Eotaxin with greater diagnosis duration (β: 0.28; 95% CI: 0.16, 0.41; adj. p = 0.012; linear regression) (Figure S6). Baseline CSF inflammatory biomarker concentrations were not associated with subsequent clinical decline/AD progression (Table S4). For neurodegenerative biomarkers in CSF, there were no associations between baseline demographic/AD characteristics and biomarker concentrations after multiple testing adjustment (Figure S7). In contrast to plasma biomarkers, CSF neurodegenerative/neuroinflammatory biomarkers were not associated with subsequent clinical decline/AD progression (Table S5).

### Incident delirium is associated with clinical decline over 18-months in mild-moderate AD

Next, we examined whether Systemic Inflammatory Events (SIEs), infections, delirium and other adverse events/hospitalisations were associated with AD progression. Clinical data was available from all 510 participants. Based on review of adverse events logs by study physicians, one-third of participants (186/510; 36.5%) experienced one or more SIE, most (89.3%) of which were infections of the respiratory or urinary tract. Unexpected hospitalisations occurred in one-sixth (16.86%; 86/510), any adverse event in 84.7% (432/510) and serious adverse events in 17.7% (90/510).

Based on the FAM-CAM, incident delirium was deemed likely in one-tenth of the study population (57/510; 11.2%), just-under half of whom had multiple episodes (4.90%; 25/510). Characteristics of participants experiencing at least one episode of delirium in comparison to those not experiencing delirium are provided in Table 4. Of note, those experiencing one or more episodes of delirium had a significantly greater diagnosis duration and significantly greater baseline AD severity on all three clinical assessments (Table 4).

**Table 4:** Characteristics of study participants by delirium status.

	No Delirium (N = 453)	Delirium (N = 57)	Statistical Test
Age (Years), Mean (SD)	74.0 (8.3)	74.4 (7.9)	t = −0.14, p = 0.56
Sex			
Female, n (%)	285 (62.9%)	31 (54.4%)	
Male, n (%)	168 (37.1%)	26 (45.6%)	χ^2^ = 1.56, p = 0.21
Body Mass Index (kg/m^2^), Mean (SD)	25.4 (4.3)	25.7 (3.7)	t = −0.45, p = 0.68
Education, Age Finished, Median (IQR)	16 (14–18)	16 (14–18)	z = −0.88, 0.38
Adjusted Comorbidity Index, Median (IQR)	0 (0–1)	0 (0–1)	z = −1.19, 0.23
Diagnosis Duration (Years), Median (IQR)	1.0 (0.3–2.2)	1.6 (0.7–2.5)	z = −2.23, p = 0.03
Alzheimer Disease Severity			
ADAS-Cog (Total), Mean (SD)	34.1 (10.4)	37.9 (11.5)	t = 2.6, p = 0.01
CDR-Sb (Total), Mean (SD)	5.1 (2.6)	6.6 (3.2)	t = 4.0, p < 0.001
DAD (Total), Mean (SD)	29.4 (7.9)	27.2 (8.0)	t = −1.86, p = 0.03
Study Group			
Nilvadipine, n (%)	229 (50.1%)	29 (50.9%)	
Placebo, n (%)	224 (49.5%)	28 (49.1%)	χ^2^ = 0.00, p = 0.96

Data were compared using t-tests, chi-square tests and Wilcoxon rank sum tests as appropriate with statistical test result and accompanying p-value reported. SD: Standard Deviation; IQR: Interquartile Range; ADAS-Cog: Alzheimer Disease Assessment Scale—Cognitive Subsection; CDR-Sb: Clinical Dementia Rating—Sum of Boxes; DAD: Disability Assessment for Dementia.

**Fig. 5:**
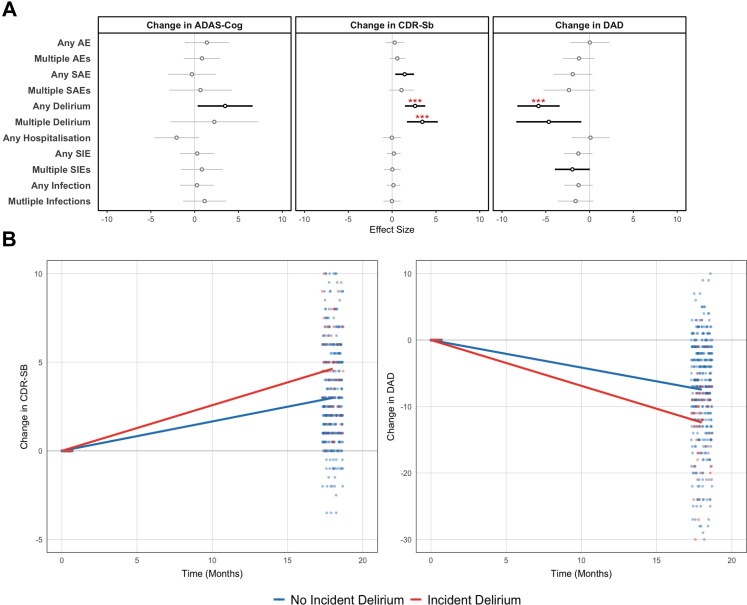
**Associations Between Incident Delirium and Cognitive Decline/Dementia Progression Over 18-Months (N** = **510).** (A) Results of linear regression (with adverse event/inflammatory event/delirium as the independent variable and change in cognitive test score/dementia severity the dependent variable) are presented as Beta Coefficients (Circles) and 95% Confidence Intervals (Lines). Non-significant results are coloured grey, with significant associations coloured black (unadjusted). Multiple-testing adjusted p-values are provided as red asterisks where ∗p < 0.05, ∗∗p < 0.01, ∗∗∗p < 0.001. (B) Associations passing the threshold for multiple testing correction are visualised against change in cognitive score at 18 months visit. ADAS-Cog: Alzheimer Disease Assessment Scale—Cognitive Subsection; CDR-Sb: Clinical Dementia Rating—Sum of Boxes; DAD: Disability Assessment for Dementia; AE: Adverse Events.

On assessment of all adverse events and after adjusting for multiple testing, only incident delirium remained associated with subsequent clinical progression (Fig. 5A). On the CDR-Sb, experiencing one or more episodes of delirium was associated with significantly greater progression by 18-months (β: 2.63; 95% CI: 1.55, 3.71; adj. p < 0.001; mixed-effects linear regression). The effect size on CDR-Sb was greater for multiple episodes of delirium (vs. no delirium) (β: 3.45; 95% CI: 1.77, 5.31; adj. p = 0.003; mixed-effects linear regression). On the DAD, incident delirium was significantly associated with greater progression by 18-months (β: −5.82; 95% CI: −8.16, −3.49; adj. p = 0.002; mixed-effects linear regression).

Findings persisted on adjusting for age, sex, BMI, education, comorbidity, diagnosis duration, baseline CDR-Sb/DAD score and study group (β: 2.44; 95% CI: 1.31, 3.58; nominal p < 0.001 on CDR-Sb for incident delirium; β: 3.72; 95% CI: 1,95, 5.48; nominal p < 0.001 on CDR-Sb for multiple vs no delirium; β: −6.38; 95% CI: −8.80, −3.96; nominal p < 0.001 on DAD for incident delirium; mixed-effects linear regression). Cognitive trajectories in those with and without incident delirium are visualised in Fig. 5B.

## Discussion

In over three-hundred individuals with established mild-moderate dementia due to AD followed for 18 months, we used high-sensitivity immunoassays to serially evaluate the relationship between systemic inflammatory biomarkers and AD progression. Baseline concentrations of chemokines and cytokines were associated with sociodemographic characteristics and remained surprisingly stable over time despite clinical progression. By contrast, blood biomarkers reflecting AD pathology (p-tau217) and neuroinflammation (GFAP) were strongly associated with subsequent clinical decline. Incident delirium–but not SIEs or infections–was associated with accelerated AD progression. However, it may equally be the case that individuals with more advanced AD may be more likely to experience delirium. Our findings have important clinical implications in the validation of emerging prognostic biomarkers in AD and in supporting delirium prevention and management efforts in those with established AD.

The lack of association between systemic inflammatory biomarkers and AD progression is perhaps surprising in the context of previous reports.^11^^,^^12^ At baseline, plasma IL-6, the “classical” marker of inflammaging, was robustly associated with age, education and BMI.^60^ IL-6 was also associated with greater disability on the DAD at baseline, although lack of association with cognitive/dementia severity on the ADAS-Cog/CDR-Sb suggests that this may be reflective of underlying sociodemographic characteristics and AD risk factors rather than dementia severity itself. A prior meta-analysis has, however, demonstrated significant associations between IL-6 and dementia severity.^61^ Our results suggest against a broad association between systemic inflammatory biomarkers and AD severity. Taken with the fact that systemic inflammatory biomarkers remained remarkably stable over time in the current study, our data suggest against a significant role for circulating chemokines/cytokines in associating with accelerated disease progression in AD. However, it must be acknowledged that by the observational nature of the current analysis, it is not possible to establish causality.

By contrast, we demonstrated strong associations between both baseline plasma p-tau217 and baseline GFAP and AD progression in established mild-moderate dementia due to AD. These findings are likely of clinical significance. For p-tau217, even after adjustment for important confounders, the middle/highest tertile of p-tau217 at baseline was associated with 2–3 points yearly decline in the ADAS-Cog with the highest tertile associated with a 2-point greater decline on the DAD. For GFAP, being in the highest tertile at baseline was associated with a one-point per year greater decline on the CDR-Sb and a near 2-point greater decline on the DAD. According to a systematic review, the Minimal Clinically Important Difference (MCID) in the ADAS-Cog is at least 2–3 points, whilst for the CDR-Sb the MCID is at least 1 point.^62^^,^^63^ Therefore, over our 18-month study duration, the data suggest that being in the highest tertile of p-tau217 and GFAP was associated with clinically-meaningful decline. Whether this continues beyond the 18-month scope of the current study cannot be extrapolated, but further long-term studies will be needed to further validate the prognostic utility of plasma p-tau217 and plasma GFAP in established AD. These associations persisted when restricting to only those with a p-tau217 level consistent with biological evidence of AD—and in many cases with larger effect sizes.

These findings are perhaps not surprising. For plasma p-tau217, previous studies have hinted at the prognostic utility of this biomarker across the AD continuum. In a study which examined stable, slow and rapid decline clusters amongst individuals with amyloid pathology cognitively unimpaired or with MCI, higher baseline plasma p-tau217 was associated with faster cognitive decline over 10 years.^29^ In another study from the Norwegian Dementia Disease Initiation and PREVENT-AD, plasma p-tau217 was associated with cognitive decline in individuals with MCI due to AD.^64^ An important role for plasma p-tau217 is further supported by BioFINDER data in predicting conversion to dementia from MCI in individuals with AD.^19^ Our findings extend these observations, suggesting that even in mild-moderate dementia stages, plasma p-tau217 may have an important role as a prognostic biomarker in AD to identify those at greatest risk of subsequent cognitive decline/clinical progression.

Whilst plasma inflammatory biomarkers were not associated with AD progression, plasma GFAP was significantly associated with clinical progression. GFAP may have an important role in early AD, whereby proteotoxicity from AD-related and other processes may result in reactive astrogliosis, which may upregulate GFAP, a cytoskeletal protein essential for maintaining astrocyte structure and function under homoeostatic conditions.^65^^,^^66^ Data also suggests that more severe astrogliosis may propagate amyloid-dependent tau phosphorylation at early disease stages.^67^ In a large study of 300 individuals from TRIAD, GFAP has been shown to be elevated in preclinical AD—amyloid positive–participants and increased in symptomatic stages of the AD continuum.^68^ In a recent study combing two separate cohorts, GFAP was shown to moderate relationships between core AD biomarkers, neurodegeneration and cognition, with higher levels of GFAP consistently relating to neurodegeneration.^69^ Again, our data extend these findings by suggesting that higher levels of GFAP may be associated with clinical decline and progression in established dementia due to AD. Again, these findings persisted on covariate adjustment and on limiting to only individuals with a plasma p-tau217 result equating to a >97.5% specificity for AD pathology.

When analysing CSF inflammatory and neurodegenerative biomarkers, only 93 of the 333 participants in the biomarker sub-studies had baseline CSF available for analysis. We did not observe associations between baseline neurodegenerative markers in CSF and subsequent cognitive decline. This may be in part due to type II error with our limited sample size and is in contrast to previously published work linking CSF p-tau181 to 1 year clinical progression in established AD^70^ as well as total-tau and ratio of total-tau to amyloid in CSF with progression in early AD.^71^ Notably, due to sample size constraints and study design, CSF biomarkers for clinically validated ATN biomarkers (for instance on Lumipulse® or Elecsys® platforms) were not carried out at study enrolment and should be considered another limitation in the interpretation of these data.

In the current study, individuals on average exhibited clinically-significant progression across all three measures. Despite this, the current study was secondary analyses of a clinical trial and so was not designed to specifically examine biomarker associations per se. Therefore, the lack of association between inflammatory biomarkers and subsequent decline may in part reflect type II error, whereby the study was not adequately powered to identify more subtle associations between inflammatory biomarkers and subsequent cognitive decline. Notwithstanding this limitation, the study was able to identify robust associations between neurodegenerative biomarkers (p-tau217 and GFAP) and subsequent decline, as well as between delirium and disease progression.

The lack of association between SIEs and subsequent cognitive decline/dementia progression is perhaps surprising, given prior evidence of an association between SIEs and disease progression.^12^ One reason for this is that SIEs in the current study were ascertained by retrospective reviews of adverse events logs which lacked information on the exact magnitude and severity of adverse events. Whilst review was conducted by geriatric medicine physicians, the lack of prospective ascertainment in a standardised fashion may have influenced our results and is a significant limitation. Further, lack of a blood sample around the time of the event to identify those events with a significant effect on systemic inflammation is also a limitation of our analysis.

Due to the study design in NILVAD, delirium was identified retrospectively using the FAM-CAM from caregivers. This may have significantly underestimated the incidence of delirium in the study participants over the 18-months, given reliance on caregiver report and by design its retrospective nature. Similarly, SIEs were identified retrospectively by review of adverse events logs, a very crude measure of ascertainment, partially mitigated by independent review by two separate physicians. Due to the nature of study reporting (logs and FAM-CAM administered at study visits), it was not possible for us to assess the temporal relationships between delirium and SIEs and the overlap between them. Thus, the episodes resulting in a recalled episode of delirium may represent the most extreme SIEs. This is a very significant limitation of the current data and whilst we did not detect an effect of SIEs on progression in the current study, future clinical studies should assess these relationships prospectively, ideally with contemporaneous medical assessment and blood sampling to further elucidate this relationship.

However, due to the inclusion of the FAM-CAM across eight study visits, we were able to ascertain events likely consistent with incident delirium in a standardised fashion. Our results suggesting accelerated clinically-meaningful progression in those experiencing delirium, whilst clinically-intuitive and perhaps unsurprising, are striking. In several prior longitudinal studies in community-dwelling older adults, delirium has been linked to greater cognitive decline over time.^72^^,^^73^ Our findings echo prior studies showing that delirium is associated with cognitive decline in individuals with dementia over and above the effect suggested by either alone.^74^^,^^75^ The magnitude of effect of nearly 3 points on the CDR-Sb for one or more episodes of incident delirium and of just-over 4 points for multiple episodes of delirium are striking and exceed MCIDs in dementia clinical trials for emerging disease modifying therapies.^63^^,^^76^^,^^77^ Thus, these findings strongly suggest that assessment for delirium should be incorporated into all dementia clinical trials as an important confounder. Additionally, these findings underscore the importance of delirium recognition, prevention and treatment measures in older adults with established dementia.

Our study is notable for its close follow-up, including comprehensive clinical assessment for cognitive decline/dementia progression using gold-standard instruments, high rate of follow-up with plasma samples available for 258 individuals at all three timepoints over 18 months. Additionally, our use of a consistent and reliable high-sensitivity assay to measure biomarker concentrations, allowed us to accurately profile low-abundant biomarkers including low-abundant cytokines typically difficult to accurately quantify and neurodegenerative biomarkers of AD pathology, neurodegeneration and neuroinflammation.

A very important limitation in the current analyses is the use of clinical NINCDS-AD criteria and not biomarker-assisted diagnosis which is now gold-standard.^78^^,^^79^

The NILVAD trial was conducted at a time when NINCDS-AD was the gold standard. When based on clinical phenotyping alone, AD may be inaccurately diagnosed in a significant number of individuals.^80^ However, in taking advantage of the excellent performance of plasma p-tau217 as a diagnostic biomarker, we were able to restrict analyses to only those with likely AD pathology. This crucial step meant we were able to apply an established cut-off for plasma p-tau217 associated with 97.5% specificity to detect amyloid pathology, the earliest pathological hallmark of AD.^59^ Whilst plasma p-tau217 is increasingly recognised as an outstanding blood-based diagnostic biomarker in AD, our lack of contemporaneous ATN diagnoses based on CSF or Positron Emission Tomography (PET) remains a limitation of the current data. Interestingly, at this plasma p-tau217 cut-off at 97.5% specificity, over 90% of the study population had biological evidence of AD, which is noteworthy for a clinical trial compared to previous trials based on clinical criteria alone.^81^

Another important limitation in the current study is the storage of samples for eight years between processing and measurement. However, samples were stored at −80 °C in a monitored biobank for this interval with no freeze–thaw cycles and temperature-monitored shipments for sample transfer. There is evidence that cytokines such as IL-6 and TNF-α are stable over such durations, especially when not exposed to multiple freeze–thaw cycles.^82^ Stability has also been demonstrated for MCP-1 after 10 years of storage.^83^ However, given the known effect of storage time on protein concentration,^84^ this remains a significant limitation of the current data. Importantly, all samples were stored under identical conditions and so any effect would have occurred across the entire cohort. Additionally, as we used high-sensitivity assays, we were able to detect analytes in nearly all samples and so long-term storage did not influence our ability to detect these analytes in the first instance. However, it is possible that long-term storage may contribute to type II error in our lack of association between inflammatory biomarkers and disease progression in mild-moderate AD.

By design our study is observational and not causal in nature. Whilst a key unanswered question for the field is whether systemic inflammation reflects sociodemographic characteristics, directly reflects AD pathology or contributes causally to clinical decline in AD, our study cannot directly assess the directionality and causality of these relationships and remains observational in nature. A final limitation in the current analysis is the lack of replication cohort outside the NILVAD study. Whilst our study involved samples from multiple centres processed in an identical fashion, replication in an external cohort would further strengthen the findings of the current report.

In summary, using data and clinical samples from over 300 individuals with mild-moderate AD dementia and high-sensitivity immunoassays, we conducted a thorough longitudinal investigation of systemic inflammatory biomarkers in AD. Systemic inflammatory biomarkers remained stable over time and did not predict accelerated decline in AD. Rather biomarkers of neuroinflammation (GFAP) and AD pathology (p-tau217) were associated with clinically-significant decline by 18-months. Incident delirium was associated with accelerated progression, encouraging the incorporation of delirium assessments into AD clinical trials and underscoring the importance of delirium prevention and management strategies in this vulnerable cohort.

## Contributors

Conceptualisation: AHD, NMB, SPK.

Study design: AHD, NMB, SPK.

Data curation (clinical): AHD, HD, LM.

Data curation (biomarker): AHD, TK, PGF, CC.

Formal analysis: AHD, HD, LM, TK, CC.

Investigation: AHD, HD, LM, TK, PGF, CC.

Methodology: AHD, NMB, SPK.

Literature review: AHD, HD, LM, TK, CC, AOC.

Writing—original draft: AHD, HD, LM, TK, CC, AOC.

Writing—review & editing: All authors.

Data access and verification: AHD, SPK.

Supervision: SPK, NMB.

The NILVAD Study group assisted with participant recruitment, assessment and completion of the NILVAD clinical trial as per the trial protocol. All authors read and approved the final version of the manuscript.

## Data sharing statement

Due to the established protocols for the NILVAD study, the consortium agreement entered into, and Irish/European data protection law and risk of patient identification, the data accompanying the current manuscript is not publicly available. However, all requests for anonymised data will be promptly reviewed by the corresponding author (AHD) and NILVAD Principal Investigator (BL) and bulk anonymised data provided on reasonable request, providing data transfer is in agreement with EU legislation and the NILVAD ethical approval, and will be accompanied by an appropriate Materials Transfer Agreement (MTA). Requests for access to data for the current study can be directed to the corresponding author (dyera@tcd.ie).

## Declaration of interests

The NILVAD Study Group was funded under the European Commission Grant (FP7 grant; 279093) to Principal Investigator Professor Brian Lawlor. The authors have no conflicts of interest to report.

## References

[1] Abbatecola A.M., Giuliani A., Biscetti L. (2024). Circulating biomarkers of inflammaging and Alzheimer's disease to track age-related trajectories of dementia: can we develop a clinically relevant composite combination?. Ageing Res Rev.

[2] Walker K.A., Le Page L.M., Terrando N., Duggan M.R., Heneka M.T., Bettcher B.M. (2023). The role of peripheral inflammatory insults in Alzheimer's disease: a review and research roadmap. Mol Neurodegener.

[3] Wichmann M.A., Cruickshanks K.J., Carlsson C.M. (2014). Long-term systemic inflammation and cognitive impairment in a population-based cohort. J Am Geriatr Soc.

[4] Walker K.A., Gottesman R.F., Wu A. (2019). Systemic inflammation during midlife and cognitive change over 20 years: the ARIC Study. Neurology.

[5] Pagoni P., Korologou-Linden R.S., Howe L.D. (2022). Causal effects of circulating cytokine concentrations on risk of Alzheimer's disease and cognitive function. Brain Behav Immun.

[6] Fard M.T., Savage K.M., Stough C.K. (2022). Peripheral inflammation marker relationships to cognition in healthy older adults - a systematic review. Psychoneuroendocrinology.

[7] Engelhart M.J., Geerlings M.I., Meijer J. (2004). Inflammatory proteins in plasma and the risk of dementia: the rotterdam study. Arch Neurol.

[8] Brosseron F., Krauthausen M., Kummer M., Heneka M.T. (2014). Body fluid cytokine levels in mild cognitive impairment and Alzheimer's disease: a comparative overview. Mol Neurobiol.

[9] Kipinoinen T., Toppala S., Rinne J.O., Viitanen M.H., Jula A.M., Ekblad L.L. (2022). Association of midlife inflammatory markers with cognitive performance at 10-Year Follow-up. Neurology.

[10] Dyer A.H., McNulty H., Caffrey A. (2024). Low-Grade systemic inflammation is associated with domain-specific cognitive performance and cognitive decline in older adults: data from the TUDA study. Neurobiol Aging.

[11] Morgan A.R., Touchard S., Leckey C. (2019). Inflammatory biomarkers in Alzheimer's disease plasma. Alzheimers Dement.

[12] Holmes C., Cunningham C., Zotova E. (2009). Systemic inflammation and disease progression in Alzheimer disease. Neurology.

[13] Lee W.J., Liao Y.C., Wang Y.F., Lin I.F., Wang S.J., Fuh J.L. (2018). Plasma MCP-1 and cognitive decline in patients with Alzheimer's disease and mild cognitive impairment: a two-year Follow-up study. Sci Rep.

[14] Yang H.S., Zhang C., Carlyle B.C. (2022). Plasma IL-12/IFN-γ axis predicts cognitive trajectories in cognitively unimpaired older adults. Alzheimers Dement.

[15] Swardfager W., Lanctôt K., Rothenburg L., Wong A., Cappell J., Herrmann N. (2010). A meta-analysis of cytokines in Alzheimer's disease. Biol Psychiatry.

[16] Foley K.E., Winder Z., Sudduth T.L. (2024). Alzheimer's disease and inflammatory biomarkers positively correlate in plasma in the UK-ADRC cohort. Alzheimers Dement.

[17] Ashton N.J., Brum W.S., Di Molfetta G. (2024). Diagnostic accuracy of a plasma phosphorylated tau 217 immunoassay for alzheimer disease pathology. JAMA Neurol.

[18] Arranz J., Zhu N., Rubio-Guerra S. (2024). Diagnostic performance of plasma pTau(217), pTau(181), Aβ(1-42) and Aβ(1-40) in the LUMIPULSE automated platform for the detection of Alzheimer disease. Alzheimers Res Ther.

[19] Mattsson-Carlgren N., Janelidze S., Palmqvist S. (2020). Longitudinal plasma p-tau217 is increased in early stages of Alzheimer's disease. Brain.

[20] Mullard A. (2023). NfL makes regulatory debut as neurodegenerative disease biomarker. Nat Rev Drug Discov.

[21] Aschenbrenner A.J., Li Y., Henson R.L. (2022). Comparison of plasma and CSF biomarkers in predicting cognitive decline. Ann Clin Transl Neurol.

[22] Gafson A.R., Barthélemy N.R., Bomont P. (2020). Neurofilaments: neurobiological foundations for biomarker applications. Brain.

[23] Chatterjee P., Pedrini S., Stoops E. (2021). Plasma glial fibrillary acidic protein is elevated in cognitively normal older adults at risk of Alzheimer's disease. Transl Psychiatry.

[24] Chiotis K., Johansson C., Rodriguez-Vieitez E. (2023). Tracking reactive astrogliosis in autosomal dominant and sporadic Alzheimer's disease with multi-modal PET and plasma GFAP. Mol Neurodegener.

[25] Cicognola C., Janelidze S., Hertze J. (2021). Plasma glial fibrillary acidic protein detects Alzheimer pathology and predicts future conversion to Alzheimer dementia in patients with mild cognitive impairment. Alzheimers Res Ther.

[26] Kim K.Y., Shin K.Y., Chang K.A. (2023). GFAP as a potential biomarker for Alzheimer's disease: a systematic review and meta-analysis. Cells.

[27] Pereira J.B., Janelidze S., Smith R. (2021). Plasma GFAP is an early marker of amyloid-β but not tau pathology in Alzheimer's disease. Brain.

[28] Palmqvist S., Whitson H.E., Allen L.A. (2025). Alzheimer's Association Clinical Practice Guideline on the use of blood-based biomarkers in the diagnostic workup of suspected Alzheimer's disease within specialized care settings. Alzheimers Dement.

[29] Kirsebom B.E., Gonzalez-Ortiz F., Vigneswaran S. (2025). Repeated plasma p-tau217 measurements to monitor clinical progression heterogeneity. Alzheimers Dement.

[30] Wu D., Milutinovic M.D., Walt D.R. (2015). Single molecule array (Simoa) assay with optimal antibody pairs for cytokine detection in human serum samples. Analyst.

[31] Posseme C., Llibre A., Charbit B. (2022). Early IFNβ secretion determines variable downstream IL-12p70 responses upon TLR4 activation. Cell Rep.

[32] Bekaddour N., Smith N., Beitz B. (2023). Targeting the chemokine receptor CXCR4 with histamine analog to reduce inflammation in juvenile arthritis. Front Immunol.

[33] Lopez-Rodriguez A.B., Hennessy E., Murray C.L. (2021). Acute systemic inflammation exacerbates neuroinflammation in Alzheimer's disease: Il-1β drives amplified responses in primed astrocytes and neuronal network dysfunction. Alzheimers Dement.

[34] Cunningham C., Wilcockson D.C., Campion S., Lunnon K., Perry V.H. (2005). Central and systemic endotoxin challenges exacerbate the local inflammatory response and increase neuronal death during chronic neurodegeneration. J Neurosci.

[35] Cunningham C., Campion S., Lunnon K. (2009). Systemic inflammation induces acute behavioral and cognitive changes and accelerates neurodegenerative disease. Biol Psychiatry.

[36] Torvell M., Hampton D.W., Connick P., MacLullich A.M.J., Cunningham C., Chandran S. (2019). A single systemic inflammatory insult causes acute motor deficits and accelerates disease progression in a mouse model of human tauopathy. Alzheimers Dement (N Y).

[37] Kahn M.S., Kranjac D., Alonzo C.A. (2012). Prolonged elevation in hippocampal Aβ and cognitive deficits following repeated endotoxin exposure in the mouse. Behav Brain Res.

[38] Philippens I.H., Ormel P.R., Baarends G., Johansson M., Remarque E.J., Doverskog M. (2017). Acceleration of amyloidosis by inflammation in the amyloid-beta marmoset monkey model of Alzheimer's disease. J Alzheimers Dis.

[39] Lawlor B., Kennelly S., O'Dwyer S. (2014). NILVAD protocol: a European multicentre double-blind placebo-controlled trial of nilvadipine in mild-to-moderate Alzheimer's disease. BMJ Open.

[40] Lawlor B., Segurado R., Kennelly S. (2018). Nilvadipine in mild to moderate Alzheimer disease: a randomised controlled trial. PLoS Med.

[41] McKhann G., Drachman D., Folstein M., Katzman R., Price D., Stadlan E.M. (1984). Clinical diagnosis of Alzheimer's disease: report of the NINCDS-ADRDA Work Group under the auspices of Department of Health and Human Services Task Force on Alzheimer's disease. Neurology.

[42] Folstein M.F., Robins L.N., Helzer J.E. (1983). The mini-mental state examination. Arch Gen Psychiatry.

[43] Charlson M.E., Carrozzino D., Guidi J., Patierno C. (2022). Charlson comorbidity index: a critical review of clinimetric properties. Psychother Psychosom.

[44] Rosen W.G., Mohs R.C., Davis K.L. (1984). A new rating scale for Alzheimer's disease. Am J Psychiatry.

[45] Morris J.C. (1997). Clinical dementia rating: a reliable and valid diagnostic and staging measure for dementia of the Alzheimer type. Int Psychogeriatr.

[46] Gélinas I., Gauthier L., McIntyre M., Gauthier S. (1999). Development of a functional measure for persons with Alzheimer's disease: the disability assessment for dementia. Am J Occup Ther.

[47] Steis M.R., Evans L., Hirschman K.B. (2012). Screening for delirium using family caregivers: convergent validity of the Family Confusion Assessment Method and interviewer-rated Confusion Assessment Method. J Am Geriatr Soc.

[48] Meulenbroek O., O'Dwyer S., de Jong D. (2016). European multicentre double-blind placebo-controlled trial of Nilvadipine in mild-to-moderate Alzheimer's disease-the substudy protocols: NILVAD frailty; NILVAD blood and genetic biomarkers; NILVAD cerebrospinal fluid biomarkers; NILVAD cerebral blood flow. BMJ Open.

[49] Tan Z.S., Beiser A.S., Vasan R.S. (2007). Inflammatory markers and the risk of Alzheimer disease: the Framingham Study. Neurology.

[50] Schneeberger S., Kim S.J., Geesdorf M.N. (2025). Interleukin-12 signaling drives Alzheimer's disease pathology through disrupting neuronal and oligodendrocyte homeostasis. Nature Aging.

[51] Cao M., Liu J., Zhang X. (2023). IL-17A promotes the progression of Alzheimer's disease in APP/PS1 mice. Immun Ageing.

[52] Sun L., Chen C. (2025). Senescence in aging and Alzheimer’s disease. Aging Dis.

[53] Oh H.S., Le Guen Y., Rappoport N. (2025). Plasma proteomics links brain and immune system aging with healthspan and longevity. Nat Med.

[54] Zhou F., Sun Y., Xie X., Zhao Y. (2023). Blood and CSF chemokines in Alzheimer's disease and mild cognitive impairment: a systematic review and meta-analysis. Alzheimers Res Ther.

[55] Wang H., Zong Y., Zhu L., Wang W., Han Y. (2023). Chemokines in patients with Alzheimer's disease: a meta-analysis. Front Aging Neurosci.

[56] Bateman R.M., Sharpe M.D., Jagger J.E. (2016). 36th international symposium on intensive care and emergency medicine: brussels, Belgium. 15-18 March 2016. Crit Care.

[57] Morrison L., Dyer A.H., Dolphin H. (2024). Circulating Interleukin-17A is associated with executive function in middle aged adults with and without type 2 diabetes. Brain Behav Immun Health.

[58] Hawerkamp H.C., Dyer A.H., Patil N.D. (2023). Characterisation of the pro-inflammatory cytokine signature in severe COVID-19. Front Immunol.

[59] Dyer A.H., Dolphin H., O'Connor A. (2024). Performance of plasma p-tau217 for the detection of amyloid-β positivity in a memory clinic cohort using an electrochemiluminescence immunoassay. Alzheimers Res Ther.

[60] Maggio M., Guralnik J.M., Longo D.L., Ferrucci L. (2006). Interleukin-6 in aging and chronic disease: a magnificent pathway. J Gerontol A Biol Sci Med Sci.

[61] Lai K.S.P., Liu C.S., Rau A. (2017). Peripheral inflammatory markers in Alzheimer's disease: a systematic review and meta-analysis of 175 studies. J Neurol Neurosurg Psychiatry.

[62] Muir R.T., Hill M.D., Black S.E., Smith E.E. (2024). Minimal clinically important difference in Alzheimer's disease: rapid review. Alzheimers Dement.

[63] Lansdall C.J., McDougall F., Butler L.M. (2023). Establishing clinically meaningful change on outcome assessments frequently used in trials of mild cognitive impairment due to Alzheimer's disease. J Prev Alzheimers Dis.

[64] Gonzalez-Ortiz F., Kirsebom B.E., Yakoub Y. (2025). Associations between changes in levels of phosphorylated tau and severity of cognitive impairment in early alzheimer disease. Neurology.

[65] Heneka M.T., Rodríguez J.J., Verkhratsky A. (2010). Neuroglia in neurodegeneration. Brain Res Rev.

[66] Liddelow S.A., Barres B.A. (2017). Reactive astrocytes: production, function, and therapeutic potential. Immunity.

[67] Bellaver B., Povala G., Ferreira P.C.L. (2023). Astrocyte reactivity influences amyloid-β effects on tau pathology in preclinical Alzheimer's disease. Nat Med.

[68] Benedet A.L., Milà-Alomà M., Vrillon A. (2021). Differences between plasma and cerebrospinal fluid glial fibrillary acidic protein levels across the alzheimer disease continuum. JAMA Neurol.

[69] Lee S.Y., Diaz V.E., Emanuel O.M. (2025). Moderating effects of plasma glial fibrillary acidic protein along the Alzheimer's disease continuum. Alzheimers Dement.

[70] Snider B.J., Fagan A.M., Roe C. (2009). Cerebrospinal fluid biomarkers and rate of cognitive decline in very mild dementia of the Alzheimer type. Arch Neurol.

[71] Vemuri P., Wiste H.J., Weigand S.D. (2009). MRI and CSF biomarkers in normal, MCI, and AD subjects: predicting future clinical change. Neurology.

[72] Davis D.H., Muniz Terrera G., Keage H. (2012). Delirium is a strong risk factor for dementia in the oldest-old: a population-based cohort study. Brain.

[73] Richardson S.J., Davis D.H.J., Stephan B.C.M. (2021). Recurrent delirium over 12 months predicts dementia: results of the Delirium and Cognitive Impact in Dementia (DECIDE) study. Age Ageing.

[74] Davis D.H., Muniz-Terrera G., Keage H.A. (2017). Association of delirium with cognitive decline in late life: a neuropathologic study of 3 population-based cohort studies. JAMA Psychiatry.

[75] Fong T.G., Vasunilashorn S.M., Libermann T., Marcantonio E.R., Inouye S.K. (2019). Delirium and Alzheimer disease: a proposed model for shared pathophysiology. Int J Geriatr Psychiatry.

[76] Angioni D., Cummings J., Lansdall C.J. (2024). Clinical meaningfulness in Alzheimer's disease clinical trials. A report from the EU-US CTAD task force. J Prev Alzheimers Dis.

[77] Lansdall C.J., Teng E., Chague J. (2024). Care partner-informed meaningful change thresholds for the Clinical Dementia Rating-Sum of Boxes for trials of early Alzheimer's disease. Alzheimers Dement.

[78] Frisoni G.B., Festari C., Massa F. (2024). European intersocietal recommendations for the biomarker-based diagnosis of neurocognitive disorders. Lancet Neurol.

[79] Dolphin H., Dyer A.H., Morrison L., Shenkin S.D., Welsh T., Kennelly S.P. (2024). New horizons in the diagnosis and management of Alzheimer's disease in older adults. Age Ageing.

[80] Rabinovici G.D., Gatsonis C., Apgar C. (2019). Association of amyloid positron emission tomography with subsequent change in clinical management among medicare beneficiaries with mild cognitive impairment or dementia. Jama.

[81] Siemers E.R., Sundell K.L., Carlson C. (2016). Phase 3 solanezumab trials: secondary outcomes in mild Alzheimer's disease patients. Alzheimers Dement.

[82] Cipriani E., Giguère C.-É., Le Page C. (2025). Long-term storage stability of plasma TNF-α and IL-6 concentrations of psychiatric emergency patients: the Signature Biobank. Cytokine.

[83] Kavsak P.A., Newman A.M., Ko D.T., Macrae A.R., Jaffe A.S. (2010). The use of a cytokine panel to define the long-term risk stratification of heart failure/death in patients presenting with chest pain to the emergency department. Clin Biochem.

[84] Enroth S., Hallmans G., Grankvist K., Gyllensten U. (2016). Effects of long-term storage time and original sampling month on biobank plasma protein concentrations. eBioMedicine.

