# Supplementary material for: Systemic inflammation, delirium and clinical progression in mild-moderate Alzheimer disease
Source: eBioMedicine. 2026 Feb 17;125:106159. doi: 10.1016/j.ebiom.2026.106159 (PMC12988547; doi:10.1016/j.ebiom.2026.106159)
Supplement: Supplementary Tables and Figures [file mmc1.docx]

**Systemic inflammatory biomarkers and clinical progression in mild-moderate Alzheimer Disease**

Adam H Dyer et al.

Supplemental Material.


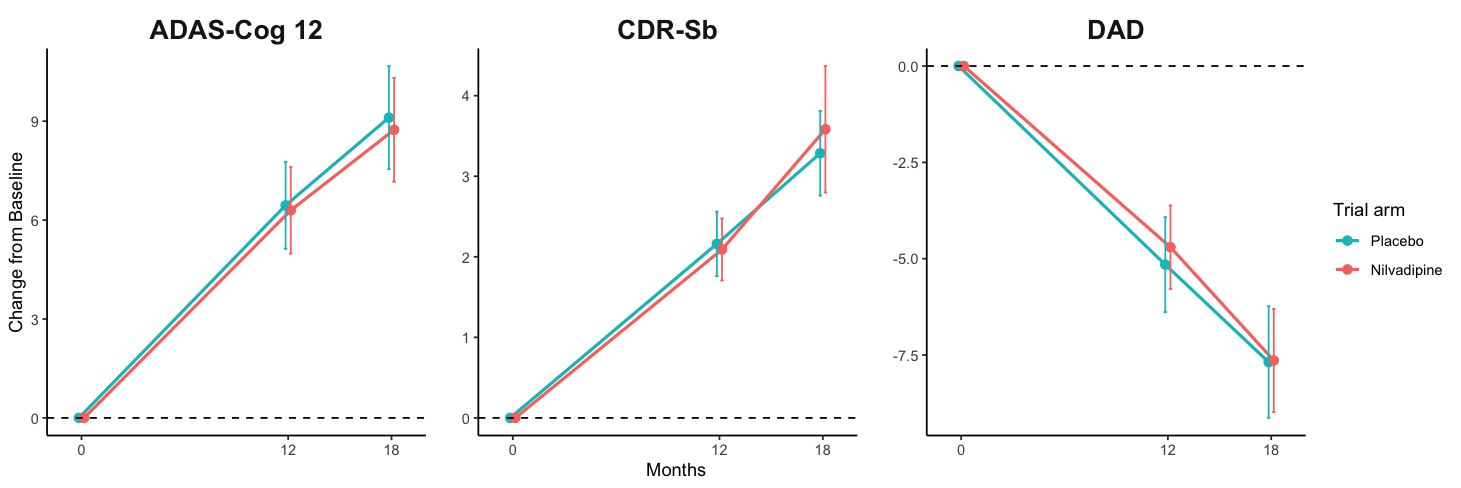


**Figure S1. Change in Dementia Severity Over Time by Study Arm.** In individuals from the NILVAD Blood and Genetic Biomarker Sub-Study (N = 333), change in Alzheimer Disease Assessment Scale – Cognitive Subsection (ADAS-CoG), Clinical Dementia Rating Scale – Sum of Boxes (CDR-Sb) and Disability Assessment for Dementia (DAD) is plotted over time. There were no significant differences in clinical progression on any of the three assessment scales by study group assessed by an Arm x Time interaction.


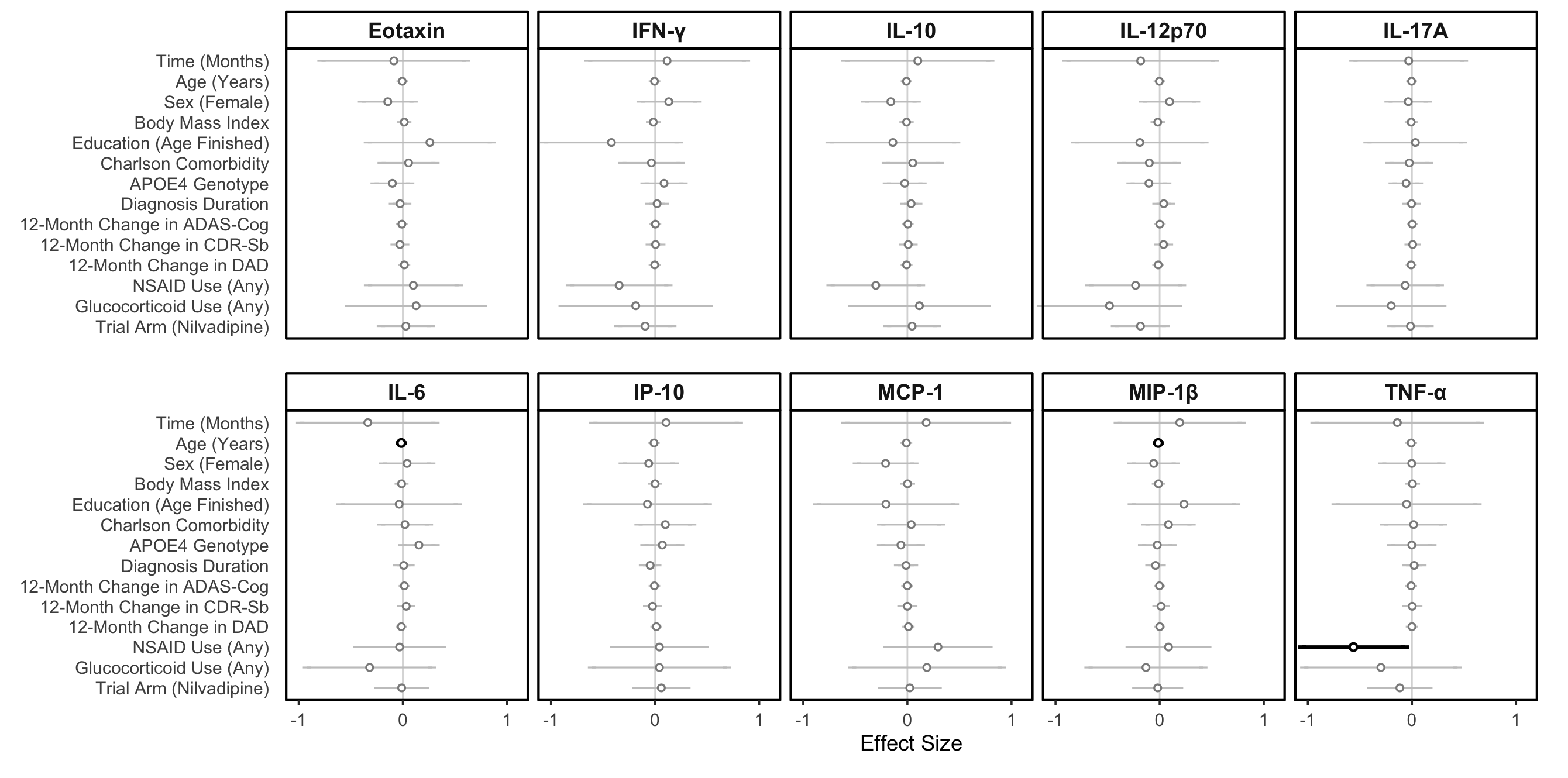


**Figure S2. Predictors of Change in Plasma Cytokine/Chemokine Concentrations at 12-Months in Mild-Moderate Alzheimer Disease.** For all ten cytokines/chemokines studied, the relationship between demographic/medical/Alzheimer Disease related factors and 12-month change in cytokine/chemokine level was explored using univariate linear regression. Results are graphed as β-coefficients (coloured dots) with 95% confidence intervals (in bold black lines). Red asterisks indicate significance levels (*p<0.05, **p<0.01, ***p<0.001). Note the top predictor in each model is Time (in Months) to test whether chemokine/cytokine concentrations significantly changed over time in study participants.


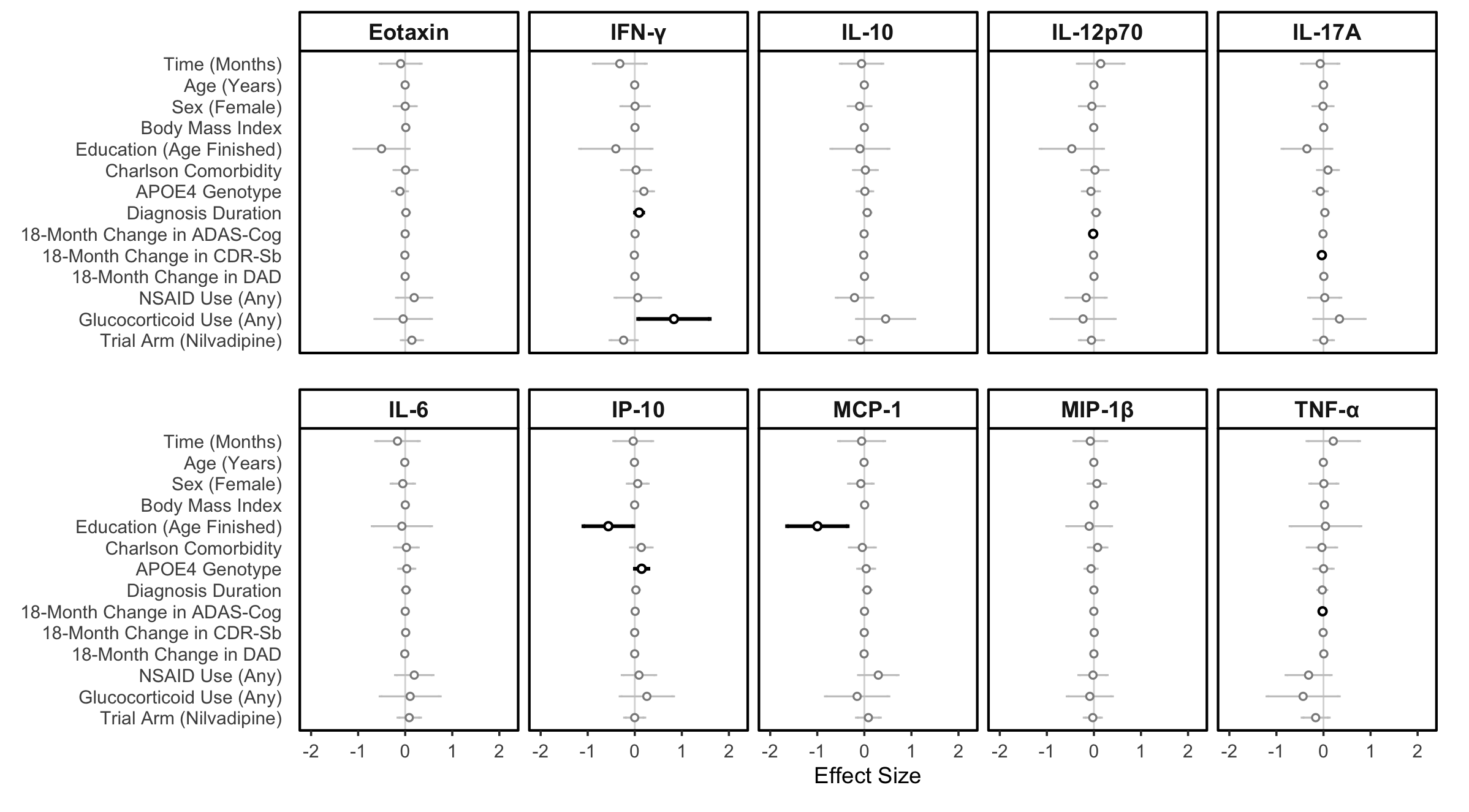


**Figure S3. Predictors of Change in Plasma Cytokine/Chemokine Concentrations at 18-Months in Mild-Moderate Alzheimer Disease.** For all ten cytokines/chemokines studied, the relationship between demographic/medical/Alzheimer Disease related factors and 18-month change in cytokine/chemokine level was explored using univariate linear regression. Results are graphed as β-coefficients (coloured dots) with 95% confidence intervals (in bold black lines). Red asterisks indicate significance levels (*p<0.05, **p<0.01, ***p<0.001). Note the top predictor in each model is Time (in Months) to test whether chemokine/cytokine concentrations significantly changed over time in study participants.

*
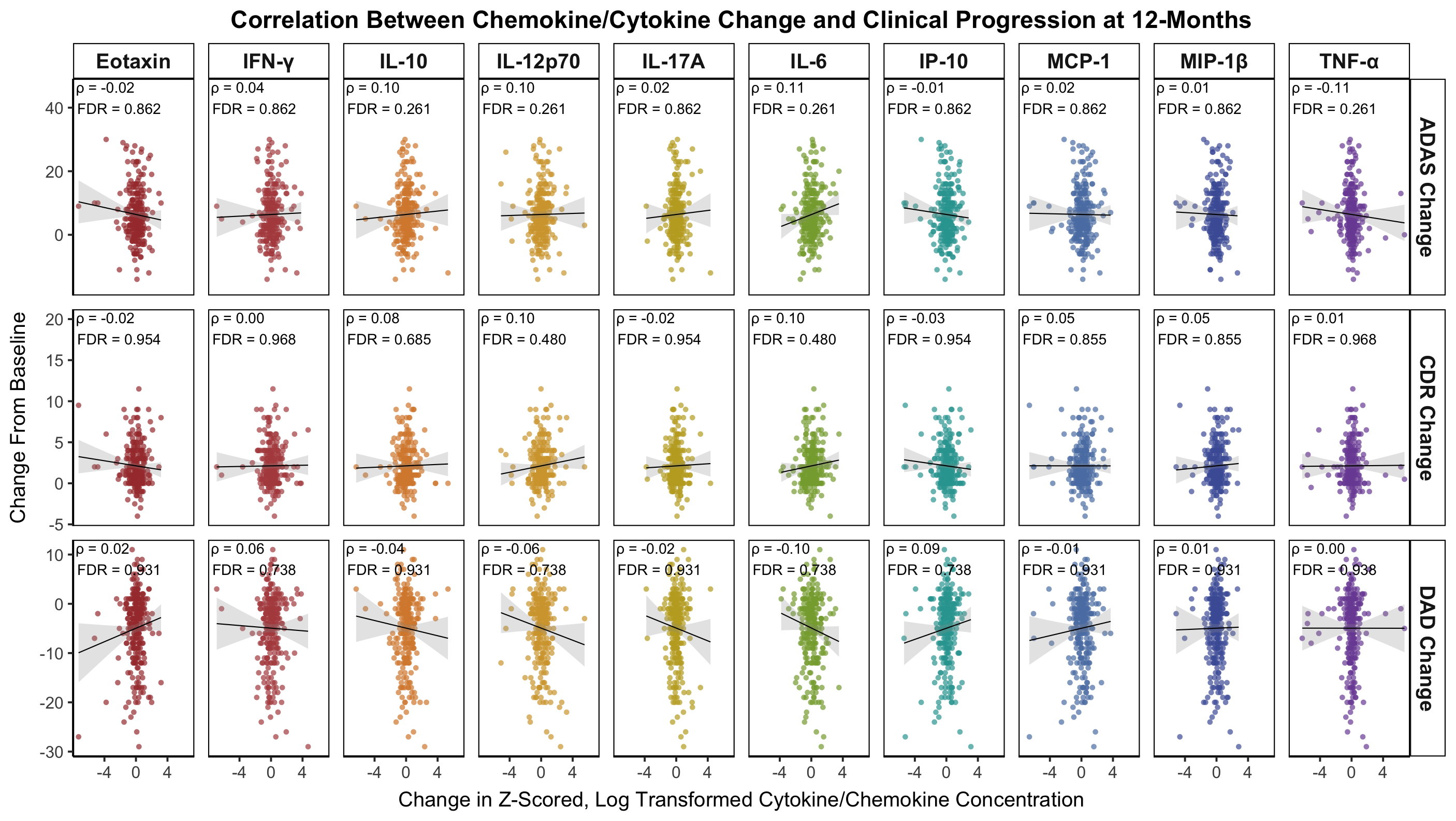
*

**Figure S4. Relationship Between Change in Chemokine/Cytokine Concentrations and Change in Alzheimer Disease Severity at 12-Months.** For all ten cytokines/chemokines studied, the change from baseline to 12 months is correlated against the change in baseline to 12 months in Alzheimer Disease severity. Results are provided as spearman’s rho (‘p’) and associated p-value corrected for multiple testing (FDR). ADAS: Alzheimer Disease Assessment Scale Cognitive Subsection; CDR: Clinical Dementia Rating Scale; DAD: Disability Assessment for Dementia.

**
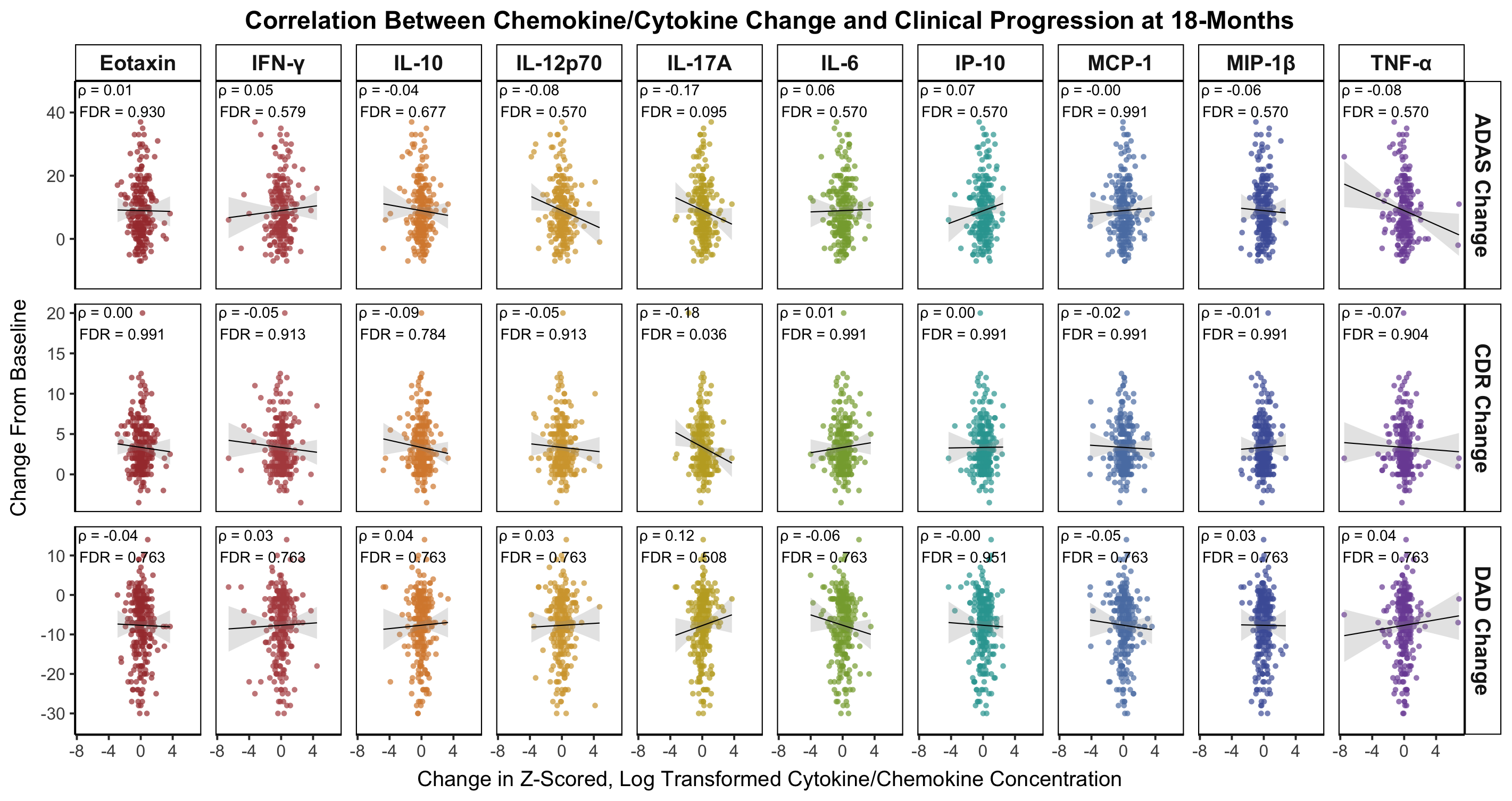
**

**Figure S5. Relationship Between Change in Chemokine/Cytokine Concentrations and Change in Alzheimer Disease Severity at 18-Months.** For all ten cytokines/chemokines studied, the change from baseline to 18 months is correlated against the change in baseline to 18 months in Alzheimer Disease severity. Results are provided as spearman’s rho (‘p’) and associated p-value corrected for multiple testing (FDR). ADAS: Alzheimer Disease Assessment Scale Cognitive Subsection; CDR: Clinical Dementia Rating Scale; DAD: Disability Assessment for Dementia

*
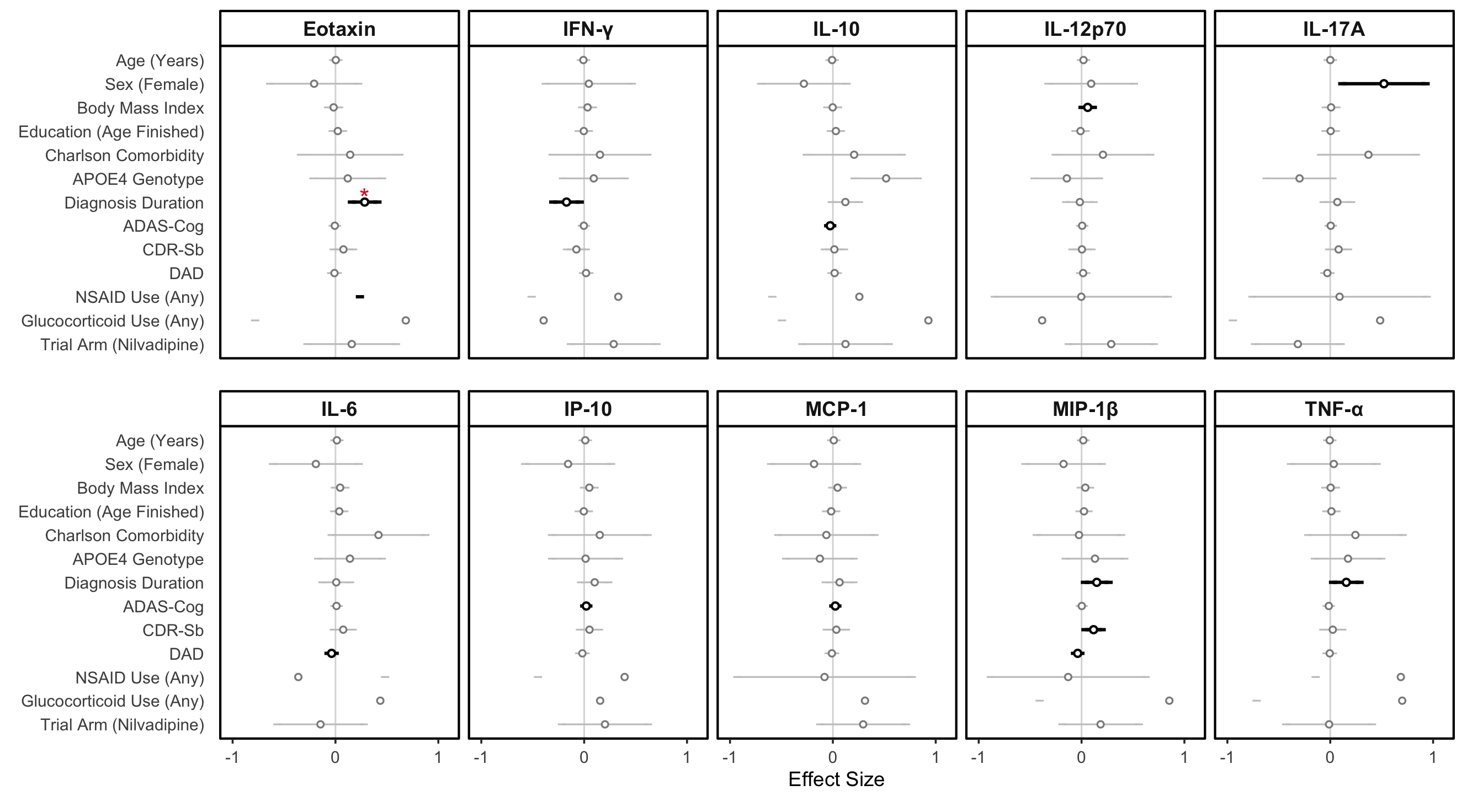
*

**Figure S6. Predictors of Cerebrospinal Fluid (CSF) Cytokine/Chemokine Concentration.** For all ten cytokines/chemokines studied, the relationship between demographic/medical/Alzheimer Disease related factors was explored using univariate linear regression. Results are graphed as β-coefficients (coloured dots) with 95% confidence intervals (in bold black lines). Red asterisks indicate significance levels (*p<0.05, **p<0.01, ***p<0.001). *APOE4: apolipoprotein E4. ADAS-Cog: Alzheimer’s Disease Assessment Scale – Cognitive Subsection; CDR-Sb: Clinical Dementia Rating Scale – Sum of Boxes, DAD: Disability Assessment for Dementia; NSAID: Non-Steroidal Anti-Inflammatory*

*
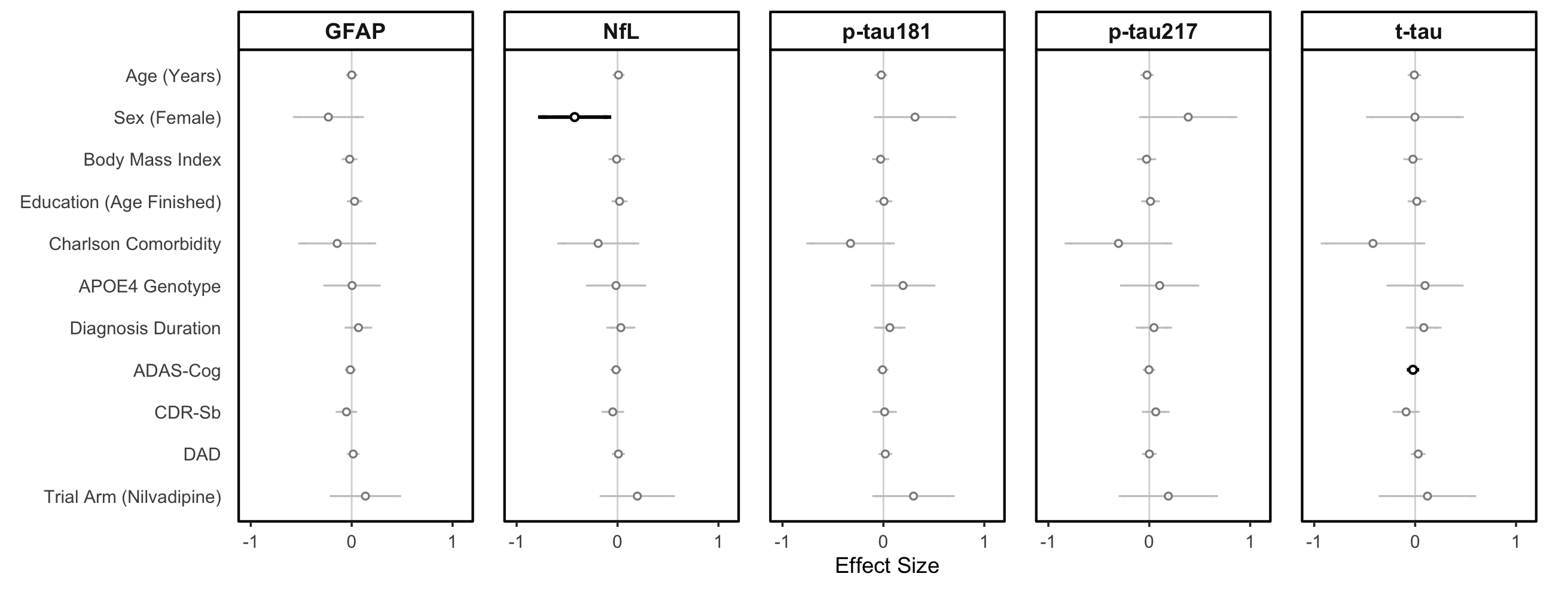
*

**Figure S7. Predictors of CSF Neurodegenerative and Neuroinflammatory Biomarker Concentrations (n = 92).** For each neurodegenerative/neuroinflammatory biomarker studied, the relationship between demographic/medical/Alzheimer Disease related factors was explored using univariate linear regression. Results are graphed as β-coefficients (coloured dots) with 95% confidence intervals (in bold black lines). Red asterisks indicate significance levels (*p<0.01). *APOE4: apolipoprotein E4. ADAS-Cog: Alzheimer’s Disease Assessment Scale – Cognitive Subsection; CDR-Sb: Clinical Dementia Rating Scale – Sum of Boxes, DAD: Disability Assessment for Dementia; NSAID: Non-Steroidal Anti-Inflammatory* *Drug. GFAP: Glial Fibrillary Acidic Protein; NfL: Neurofilament Light; t-tau: total tau; p-tau181: phosphorylated-tau181; p-tau217: phosphorylated-tau217.*

| **Country** | **Site** | **Ethics Committee** | **Reference Number(s)** |
| --- | --- | --- | --- |
| Ireland | St James’s Hospital, Dublin, Ireland | SJH/AMNCH Research Ethics Committee | 2013/13/02; VHP201268 – SA3; 2014/List 5 |
|  | St Finbarr’s Hospital, Cork, Ireland |  |  |
| United Kingdom | Institute of Psychiatry, King’s College London | NRES Committee London – Harrow | 12/LO/1903 |
| France | CHU Amiens | Comité de Protection des Personnes Nord Ouest III | CPP Ref: 2012-40 |
|  | CH Bethune |  |  |
|  | CHU Caen |  |  |
|  | CH Calais |  |  |
|  | CH Saint-Philibert, GHICL |  |  |
|  | CHRU Lille |  |  |
|  | CH Lens |  |  |
| Greece | Pagageorgiou General Hospital | Scientific Council of Papanikolaou Hospital Thessaloniki | 122/00-03/12 |
|  | Papanikolaou General Hospital of Thessaloniki |  |  |
|  | AXEPA University General Hospital |  |  |
| Netherlands | Rijnstate Hospital, Arnhem | Radboud universitair medisch centrum Concernstaf Kwaliteit en Veiligheid Commissie Mensgebonden Onderzoek Regio Arnhem-Nijmegen | NL40269.091.12; METC number: 2012/508 |
|  | Academic Hospital, Maastricht |  |  |
|  | Radboud University Medical Centre, Nijmegen |  |  |
| Hungary | University of Szeged | Medical Research Council Ethics Committee for Clinical Pharmacology (KFEB) | 11637-0/2013-EKL; 38989-2/2013; 20529-0/2014-EKL |
| Italy | IRCCS Centro san Giovanni di Dio-Fatebenefratelli Brescia | C E I O C – Comitato Etico Istituzioni Ospedaliere Cattoliche | 33/2013; 22/2014 |
|  | IRCCS Multimedica Castellanza | Comitato Etico IRCCS MultiMedica | N. 1 20/1 2012 Neurologico |
|  | IRCSS AOU San Martino Genoa | Comitato Etico dell’Aienda Ospedaliera Universitaria S. Martino di Genova  &  Comitato Etico Regione Liguria | 33 – 24/05/2013; 9 – 13/11/2013; 23 – 16/04/2014 |
|  | Fondazione Don Gnocchi, Milan | Comitato Etico Fondazione Don Carlo Gnocchi | 12.15/10/2013; 3.12/02/2014 |
| Sweden | Sahlgrenska University Hospital / Mölndal (SU/Mölndal) | Regionala etikprövningsnämnden i Göteborg | Dnr 933-12 |
| Germany | Klinik für Psychiatrie und Psychotherapie II, Universität Ulm, Bezirkskrankenhaus Günzburg | Bayerische Landesärztekammer – Ethik-Kommission | Ethik-Kommission Nr. **12120** |

**Table S1. Ethics Approval Committees and Reference Numbers for the NILVAD Study.** Data are provided for each of the 23 Study Sites across 9 European Countries participating in the NILVAD Study.

| **Analyte** | **LLOQ** | **ULOQ** | **CV Inter-** | **CV intra-** | **RANGE**  **(Plasma)** | **Detectable**  **(Plasma)** | **RANGE (CSF)** | **Detectable**  **(CSF)** |
| --- | --- | --- | --- | --- | --- | --- | --- | --- |
| **IFN-γ (S-PLEX)** | 10 fg/mL | 40,440 fg/mL | 16.7% | 7.3% | 38-56,000 fg/mL | 870/870 | 0.5-1,791 fg/mL | 140/143 |
| **IL-10 (S-PLEX)** | 43 fg/mL | 167,760 fg/mL | 24.2% | 10.0% | 51-81,278 fg/mL | 869/870 | 2-618 fg/mL | 142/143 |
| **IL-12p70 (S-PLEX)** | 123 fg/mL | 495,910 fg/mL | 24.9% | 7.5% | 12-4,158 fg/mL | 869/870 | 1-439 fg/mL | 108/143 |
| **IL-17a (S-PLEX)** | 55 fg/mL | 215,026 fg/mL | 14.6% | 7.1% | 58-30,965 fg/mL | 870/870 | 0.7-144 fg/mL | 134/143 |
| **IL-6 (S-PLEX)** | 12 fg/mL | 39,696 fg/mL | 15.1% | 7.2% | 436-97,145 fg/mL | 870/870 | 1124-43240 fg/mL | 143/143 |
| **TNF-α (S-PLEX)** | 21 fg/mL | 89,853 fg/mL | 16.8% | 6.2% | 22-2,283 fg/mL | 864/870 | 2-212 fg/mL | 141/143 |
| **Eotaxin (V-PLEX)** | 1.5 pg/mL | 1821 pg/mL | 15.3% | 5.4% | 35-1,067 pg/mL | 866/870 | 0.4-65.5pg/mL | 142/143 |
| **IP-10 (V-PLEX)** | 0.7 pg/mL | 2817 pg/mL | 10.7% | 5.6% | 48-4,200 pg/mL | 866/870 | 25-2349 pg/mL | 143/143 |
| **MCP-1 (V-PLEX)** | 0.1 pg/mL | 547 pg/mL | 9.9% | 5.2% | 17-605 pg/mL | 866/870 | 17-1307 pg/mL | 142/143 |
| **MIP-1β (V-PLEX)** | 0.5 pg/mL | 1164 pg/mL | 15.0% | 4.2% | 18-595 pg/mL | 866/870 | 3-137 pg/mL | 143/143 |

**Table S2. Performance of Multiplex Cytokine/Chemokine Measurements in Plasma (N = 870) and Cerebrospinal Fluid Samples (N = 143)**. Analytes were measured across 16 electrochemiluminescence plates. For IFN-γ, IL-10, IL-12p70, IL-17A and IL-6 analytes were measured in undiluted plasma/CSF a custom MSD S-PLEX multi-plex ultra-sensitive immunoassay kit. TNF-α was measured in undiluted plasma/CSF using an MSD S-PLEX single-plex ultra-sensitive immunoassay kit. For Eotaxin, IP-10, MCP-1 and MIP-1β, plasma and CSF were diluted 1:2 and measured using a custom MSD S-PLEX multi-plex validated standard sensitivity immunoassay kit. Results were read on an MSD Quick Plex™ 120mm instrument. The Lower Limit of Quantification (LLOQ) refers to the lowest point of the standard curve where duplicates had an intra-assay Coefficient of Variation (CV) of <20%.

| **Analyte** | **LLOQ** | **ULOQ** | **CV Inter** | **CV intra** | **RANGE**  **(Plasma)** | **Detectable (plasma)** | **RANGE (CSF)** | **Detectable (CSF)** |
| --- | --- | --- | --- | --- | --- | --- | --- | --- |
| **GFAP (S-PLEX)** | 214 fg/mL | 788,087 fg/mL | 7.2% | 3.5% | 15-337,313 fg/mL | 333/333 | 270-497,656 pg/mL | 93/93 |
| **NfL (S-PLEX)** | 49 fg/mL | 209,867 fg/mL | 18.9% | 9.3% | 509-1,018,845 fg/mL | 333/333 | 49-269,230 pg/mL | 93/93 |
| **T-tau(S-PLEX)** | 48 fg/mL | 209,867 fg/mL | 13.1% | 6.0% | 2-469,962 fg/mL | 333/333 | 21-33,882 pg/mL | 93/93 |
| **p-tau181(S-PLEX)** | 13 fg/mL | 208,6849 fg/mL | 17.2% | 5.7% | 143-14,839 fg/mL | 333/333 | 4.3-263 pg/mL | 93/93 |
| **p-tau217(S-PLEX)** | 872 fg/mL | 374,5818 fg/mL | 9.3% | 4.9% | 2387-136,151 fg/mL | 333/333 | 46-4061 pg/mL | 93/93 |

**Table S3. Performance of Multiplex Neuroinflammatory/Neurodegenerative Measurements in Plasma (N = 328) and Cerebrospinal Fluid (CSF) Samples (N = 92)**. Analytes were measured across 6 electrochemiluminescence plates for each of p-tau181, p-tau217 and a multiplex of t-tau/GFAP/NfL. Analytes were measured in undiluted plasma using singleplex p-tau181, p-tau217 and multiplex GFAP/NfL/t-tau kits. For the multiplex kit, CSF samples were diluted 1:20. At the end of each assay, plates were read on an MSD Quick Plex™ 120mm instrument. The Lower Limit of Quantification (LLOQ) refers to the lowest point of the standard curve where duplicates had an intra-assay Coefficient of Variation (CV) of <20% and the Upper Limit of Quantification (ULOQ) the highest point on the curve where duplicates had a CV of 20%. Inter-assay CV was calculated from a plasma pool of 16 donors as above, distributed on 6 spots in each of the 6 plates. Intra-assay CV was calculated from the readings of all duplicates on each plate. *GFAP: Glial Fibrillary Acidic Protein; NfL: Neurofilament Light; t-tau: total tau; p-tau181: phosphorylated-tau181; p-tau217: phosphorylated-tau217; fg/mL: femtogram per millilitre; pg/mL: picogram per millilitre*

| **Outcome** | **Predictor** | **β Coeff.** | **Lower CI** | **Upper CI** | **P-Value** |
| --- | --- | --- | --- | --- | --- |
| Change in ADAS-Cog | *CSF IFN-γ* Time* | -0.15 | -0.72 | 0.41 | 0.59 |
|  | *CSF IL-10* Time* | 0.02 | -0.47 | 0.50 | 0.95 |
|  | *CSF IL-12p70* Time* | -0.07 | -0.58 | 0.43 | 0.77 |
|  | *CSF IL-17A* Time* | 0.18 | -0.31 | 0.67 | 0.47 |
|  | *CSF IL-6* Time* | 0.19 | -0.32 | 0.70 | 0.46 |
|  | *CSF TNF-α* Time* | 0.09 | -0.40 | 0.57 | 0.73 |
|  | *CSF Eotaxin* Time* | 0.00 | -0.48 | 0.47 | 0.99 |
|  | *CSF IP-10* Time* | 0.11 | -0.38 | 0.59 | 0.67 |
|  | *CSF MCP-1* Time* | 0.17 | -0.31 | 0.66 | 0.48 |
|  | *CSF MIP-β* Time* | 0.30 | -0.18 | 0.79 | 0.21 |
| Change in CDR-Sb | *CSF IFN-γ* Time* | -0.09 | -0.33 | 0.16 | 0.48 |
|  | *CSF IL-10* Time* | -0.01 | -0.22 | 0.20 | 0.94 |
|  | *CSF IL-12p70* Time* | 0.00 | -0.22 | 0.23 | 0.97 |
|  | *CSF IL-17A* Time* | 0.03 | -0.18 | 0.24 | 0.78 |
|  | *CSF IL-6* Time* | 0.08 | -0.15 | 0.30 | 0.50 |
|  | *CSF TNF-α* Time* | 0.01 | -0.21 | 0.22 | 0.94 |
|  | *CSF Eotaxin* Time* | 0.02 | -0.19 | 0.23 | 0.84 |
|  | *CSF IP-10* Time* | 0.05 | -0.17 | 0.26 | 0.67 |
|  | *CSF MCP-1* Time* | 0.04 | -0.18 | 0.25 | 0.74 |
|  | *CSF MIP-β* Time* | 0.10 | -0.11 | 0.31 | 0.35 |
| Change in DAD | *CSF IFN-γ* Time* | 0.15 | -0.32 | 0.63 | 0.52 |
|  | *CSF IL-10* Time* | 0.13 | -0.28 | 0.53 | 0.55 |
|  | *CSF IL-12p70* Time* | 0.21 | -0.22 | 0.64 | 0.33 |
|  | *CSF IL-17A* Time* | 0.10 | -0.31 | 0.51 | 0.63 |
|  | *CSF IL-6* Time* | 0.03 | -0.40 | 0.45 | 0.91 |
|  | *CSF TNF-α* Time* | 0.07 | -0.34 | 0.48 | 0.75 |
|  | *CSF Eotaxin* Time* | 0.13 | -0.26 | 0.53 | 0.51 |
|  | *CSF IP-10* Time* | 0.04 | -0.38 | 0.45 | 0.87 |
|  | *CSF MCP-1* Time* | 0.04 | -0.37 | 0.45 | 0.84 |
|  | *CSF MIP-β* Time* | 0.03 | -0.38 | 0.44 | 0.89 |

**Table S4***.* **Baseline Cerebrospinal Fluid (CSF) Cytokine/Chemokine Concentrations were Not Associated with Clinical Progression in Mild-Moderate Alzheimer Disease**. β-coefficients (‘β-COEFF’) give results of mixed-effects linear regression models (*cytokine/chemokine concertation*Time* fixed effect). The lower/upper bounds of 95% Confidence Intervals are provided as ‘LOWER CI’/’UPPER CI’*. ADAS-Cog: Alzheimer’s Disease Assessment Scale – Cognitive Subsection; CDR-Sb: Clinical Dementia Rating Scale – Sum of Boxes, DAD: Disability Assessment for Dementia.*

| **Outcome** | **Predictor** | **β Coeff.** | **Lower CI** | **Upper CI** | **P-Value** |
| --- | --- | --- | --- | --- | --- |
| Change in ADAS-Cog | *CSF p-tau181* Time* | 0.09 | -0.02 | 0.20 | 0.118 |
|  | *CSF p-tau217* Time* | 0.07 | -0.04 | 0.18 | 0.243 |
|  | *CSF t-tau* Time* | -0.10 | -0.20 | 0.01 | 0.087 |
|  | *CSF GFAP* Time* | 0.00 | -0.12 | 0.11 | 0.990 |
|  | *CSF NfL* Time* | 0.02 | -0.09 | 0.14 | 0.703 |
| Change in CDR-Sb | *CSF p-tau181* Time* | 0.00 | -0.06 | 0.05 | 0.922 |
|  | *CSF p-tau217* Time* | -0.01 | -0.07 | 0.04 | 0.653 |
|  | *CSF t-tau* Time* | -0.04 | -0.09 | 0.02 | 0.163 |
|  | *CSF GFAP* Time* | -0.02 | -0.07 | 0.04 | 0.512 |
|  | *CSF NfL* Time* | -0.02 | -0.07 | 0.04 | 0.589 |
| Change in DAD | *CSF p-tau181* Time* | -0.06 | -0.15 | 0.03 | 0.180 |
|  | *CSF p-tau217* Time* | -0.04 | -0.13 | 0.05 | 0.351 |
|  | *CSF t-tau* Time* | 0.09 | 0.00 | 0.18 | 0.319 |
|  | *CSF GFAP* Time* | 0.02 | -0.07 | 0.11 | 0.703 |
|  | *CSF NfL* Time* | 0.03 | -0.06 | 0.13 | 0.464 |

**Table S5. Association Between Baseline CSF Neurodegenerative Biomarkers and Alzheimer Disease Progression Over 18 Months in the NILVAD Study.** Mixed-effects linear regressions were performed with natural log-transformed, z-scored *biomarker concentration*Time (in months)* as the predictor variable with change in cognitive test (ΔADAS-Cog/ΔCDR-Sb/ΔDAD) as the dependent variable. The above take presents the beta (β) coefficient, and 95% confidence intervals (‘LOWER CI’/’UPPER CI’) for the *biomarker*Time interaction* Term. *CSF: Cerebrospinal Fluid.* *GFAP: Glial Fibrillary Acidic Protein; NfL: Neurofilament Light; t-tau: total tau; p-tau181: phosphorylated-tau181; p-tau217: phosphorylated-tau217. ADAS-Cog: Alzheimer Disease Assessment Scale, Cognitive Subsection; CDR-Sb: Clinical Dementia Rating-Sum of Boxes; DAD: Disability Assessment for Dementia.*
