# Supplementary material for: Systemic inflammation, delirium and clinical progression in mild-moderate Alzheimer disease
Source: eBioMedicine. 2026 Feb 17;125:106159. doi: 10.1016/j.ebiom.2026.106159 (PMC12988547; doi:10.1016/j.ebiom.2026.106159)
Supplement: Supplementary Material [file mmc2.docx]

# NILVAD Study Group – Author List

| **First Name(s)** | **Surnames** |
| --- | --- |
| Brian | Lawlor |
| Ricardo | Segurado |
| Sean | Kennelly |
| Marcel G. M. | Olde Rikkert |
| Robert | Howard |
| Florence | Pasquier |
| Anne | Börjesson-Hanson |
| Magda | Tsolaki |
| Ugo | Lucca |
| D. William | Molloy |
| Robert | Coen |
| Matthias W. | Riepe |
| János | Kálmán |
| Rose Anne | Kenny |
| Fiona | Cregg |
| Sarah | O’Dwyer |
| Cathal | Walsh |
| Jessica | Adams |
| Rita | Banzi |
| Laetitia | Breuilh |
| Leslie | Daly |
| Suzanne | Hendrix |
| Paul | Aisen |
| Siobhan | Gaynor |
| Ali | Sheikhi |
| Diana G. | Taekema |
| Frans R. | Verhey |
| Raffaello | Nemni |
| Flavio | Nobili |
| Massimo | Franceschi |
| Giovanni | Frisoni |
| Orazio | Zanetti |
| Anastasia | Konsta |
| Anastasios | Orologas |
| Styliani | Nenopoulou |
| Fani | Tsolaki-Tagaraki |
| Magdolna | Pakaski |
| Olivier | Dereeper |
| Vincent | de la Sayette |
| Olivier | Sénéchal |
| Isabelle | Lavenu |
| Agnès | Devendeville |
| Gauthier | Calais |
| Fiona | Crawford |
| Michael | Mullan |
| Pauline | Aalten |
| Maria A. | Berglund |
| Jurgen A. | Claassen |
| Rianne A. | De Heus |
| Daan L. K. | De Jong |
| Olivier | Godefroy |
| Siobhan | Hutchinson |
| Aikaterini | Ioannou |
| Michael | Jonsson |
| Annette | Kent |
| Jürgen | Kern |
| Petros | Nemtsas |
| Minoa-Kalliopi | Panidou |
| Laila | Abdullah |
| Daniel | Paris |
| Angelina M. | Santoso |
| Gerrita J. | van Spijker |
| Martha | Spiliotou |
| Georgia | Thomoglou |
| Anders | Wallin |
